# Functionalization mediates heat transport in graphene nanoflakes

**DOI:** 10.1038/ncomms11281

**Published:** 2016-04-29

**Authors:** Haoxue Han, Yong Zhang, Nan Wang, Majid Kabiri Samani, Yuxiang Ni, Zainelabideen Y. Mijbil, Michael Edwards, Shiyun Xiong, Kimmo Sääskilahti, Murali Murugesan, Yifeng Fu, Lilei Ye, Hatef Sadeghi, Steven Bailey, Yuriy A. Kosevich, Colin J. Lambert, Johan Liu, Sebastian Volz

**Affiliations:** 1 Laboratoire EM2C, CNRS, CentraleSupélec, Université Paris-Saclay, Grande Voie des Vignes, 92295 Châtenay-Malabry, France; 2 SMIT Center, School of Automation and Mechanical Engineering and Institute of NanomicroEnergy, Shanghai University, 20 Chengzhong Road, Shanghai 201800, China; 3 Electronics Materials and Systems Laboratory, Department of Microtechnology and Nanoscience, Chalmers University of Technology, Kemivägen 9, SE-412 96 Gothenburg, Sweden; 4 Department of Mechanical Engineering, University of Minnesota, 111 Church Street SE, Minneapolis, Minnesota 55455, USA; 5 Quantum Technology Center, Physics Department, Lancaster University, Lancaster LA1 4YB, UK; 6 Science Department, Veterinary Medicine College, Al-Qasim Green University, Babylon, Iraq; 7 Max Planck Institute for Polymer Research, Ackermannweg 10, D-55128 Mainz, Germany; 8 Department of Biomedical Engineering and Computational Science, Aalto University, FI-00076 Aalto, Finland; 9 SHT Smart High Tech AB, Ascherbergsgatan 46, SE-411 33 Gothenburg, Sweden; 10 Department of Polymers and Composite Materials, Semenov Institute of Chemical Physics, Russian Academy of Sciences, Kosygin Street 4, 119991 Moscow, Russia

## Abstract

The high thermal conductivity of graphene and few-layer graphene undergoes severe degradations through contact with the substrate. Here we show experimentally that the thermal management of a micro heater is substantially improved by introducing alternative heat-escaping channels into a graphene-based film bonded to functionalized graphene oxide through amino-silane molecules. Using a resistance temperature probe for *in situ* monitoring we demonstrate that the hotspot temperature was lowered by ∼28 °C for a chip operating at 1,300 W cm^−2^. Thermal resistance probed by pulsed photothermal reflectance measurements demonstrated an improved thermal coupling due to functionalization on the graphene–graphene oxide interface. Three functionalization molecules manifest distinct interfacial thermal transport behaviour, corroborating our atomistic calculations in unveiling the role of molecular chain length and functional groups. Molecular dynamics simulations reveal that the functionalization constrains the cross-plane phonon scattering, which in turn enhances in-plane heat conduction of the bonded graphene film by recovering the long flexural phonon lifetime.

## Abstract (web summary)

The high thermal conductivity of graphene is considerably reduced when the two-dimensional material is in contact with a substrate. Here, the authors show that thermal management of a micro heater is improved using graphene-based films covalently bonded by amino-silane molecules to graphene oxide.

## Graphical abstract

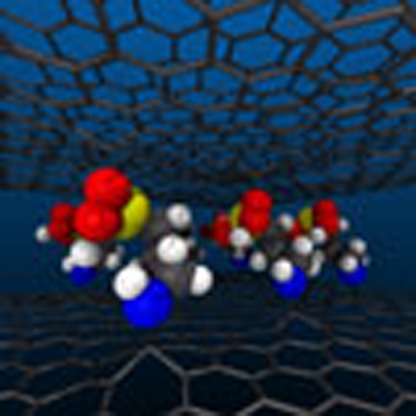

Anisotropic properties of two-dimensional (2D) layered materials make them promising in the application of next-generation electronic devices, among which graphene and few-layer graphene (FLG) have been most intensively studied for thermal management, due to their extraordinarily high in-plane thermal conductivity 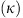
1,2,3,4,5. For instance, Yan *et al.*^6^ reported that the maximum hotspot temperature can be lowered by ∼20 °C in transistors operating at ∼13 W mm^−1^ using FLG as a heat spreader for a gallium nitride (GaN) transistor. Gao *et al.*^7^ reported that the maximum hotspot temperature decreased from 121 to 108 °C (Δ*T*=13 °C) for a heat flux of 430 W cm^−2^ after the introduction of a single-layer graphene heat spreader. Moreover, the simulations of graphene heat spreaders were also reported for silicon-on-insulator integrated circuits^8^ and three-dimensional (3D) integrated circuits^9^. The thermal conductivity of a graphene laminate film supported on substrate was also investigated and found to remain rather large^10^. However, in most practical applications, graphene/FLG will be supported by and integrated with insulators, both in electronic circuitry and heat-spreader applications^11^. Therefore, thermal energy flow will be limited both by the in-plane thermal conductivity 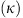 of the supported graphene/FLG and by the thermal boundary resistance (*R*) at the graphene/FLG–substrate interface^12^.

Owing to their exceedingly large surface-to-volume ratio, the properties of 2D layered materials are very sensitive to the interactions with external bodies. Indeed, when supported on an amorphous substrate, 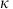 of suspended graphene decreased by almost one order of magnitude, from ∼4,000 (ref. 13) to ∼600 W m^−1^ K^−1^ (ref. 14). Such a striking discrepancy in 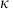 significantly limits the thermal performance of graphene/FLG in real applications. It is reported that the different behaviours are due to the strong scattering of the important heat-carrying flexural acoustic (ZA) modes^15^ to the substrate^16^. More specifically, it was identified that the phonon relaxation times of graphene ZA modes are suppressed when supported on a SiO_2_ substrate. These studies have improved our fundamental understanding in the physics behind the problem, and it was suggested that making rational choice of the substrate material^14^,^17^ and modulating its coupling to graphene^12^ may be useful to improve 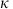 of the supported graphene/FLG.

The thermal boundary resistance (*R*) of a graphene/FLG–substrate interface is another limiting factor to their thermal performance in devices. Covalent functionalization has been proved to efficiently promote heat transfer between interfaces by introducing additional thermal pathways through the functionalizing molecules^18^,^19^,^20^,^21^,^22^,^23^,^24^,^25^,^26^,^27^,^28^,^29^,^30^,^31^,^32^,^33^. For example, self-assembled monolayers (SAMs) were used to functionalize metallic surfaces to enhance heat transport across metal–water^20^,^27^, metal–gas^28^, metal–semiconductor^21^ and metal–polymer^31^ interfaces. Functionalization was used in graphene and carbon nanotube nanocomposites to mitigate the high thermal boundary resistance between the graphene/carbon nanotube fillers and the polymer matrices^18^,^25^,^26^,^30^. Functionalized molecules also assist to align and densely pack multilayer graphene sheets and reduce the interlayer thermal resistance of graphene^25^. Recently, it was shown that plasma-functionalized graphene raised the cross-plane thermal conductance between aluminium and its substrate by a factor of two^19^. Nevertheless, the functionalization-introduced point defects will further decrease 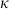 of the supported graphene/FLG, as they introduce phonon-scattering centres^25^,^32^,^33^. To correct this drawback, a robust solution that maintains the high thermal conductivity of graphene/FLG when supported, while effectively reducing the interface thermal resistance is needed.

Here we demonstrate that thermal management of a micro heater is considerably improved via introducing alternative heat-spreading channels implemented with graphene-based film (GBF) bonded to functionalized graphene oxide (FGO) through amino-silane molecules. We probed interface thermal resistance by photothermal reflectance measurements to demonstrate an improved thermal coupling due to functionalization on the graphene–graphene oxide interface and the graphene oxide–silica interface. Molecular dynamics simulations and *ab initio* calculations reveal that the functionalization constrains the cross-plane scattering of low-frequency phonons, which in turn enhances in-plane heat conduction of the bonded graphene film by recovering the long flexural phonon lifetime. Our results provide evidence that a graphene film deposited on a FGO substrate provides a very attractive platform for thermal management applications.

## Results

### Graphene-based film and graphene oxide and device

**Figure 1:**
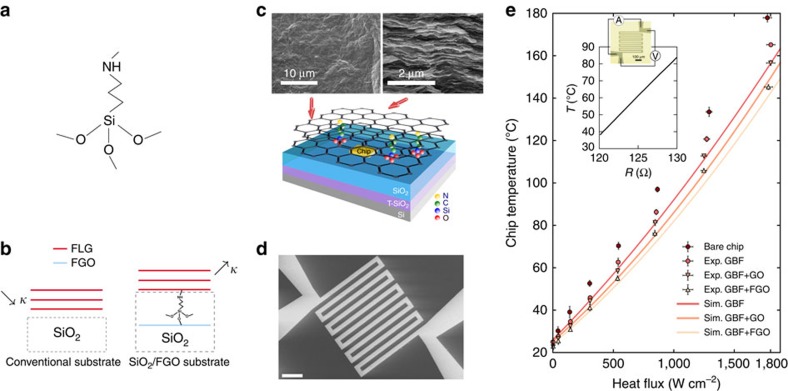
Graphene-based film on FGO as heat spreader for hotspot. (**a**) Sketch of the chemical bonds of the silane molecule. (**b**) Schematic of a graphene film on different supports. Left: conventional silica substrate. Right: the proposed silica/FGO substrate. (**c**) Schematic of the measurement set-up. The chip is embedded in the SiO_2_ substrate. T-SiO_2_ stands for thermally grown SiO_2_. Scanning electron microscopy (SEM) image of the in-plane and the cross-section of the GBF. (**d**) SEM image of the chip as the hotspot. Scale bar, 100 μm. (**e**) Measured (filled markers) and finite-element-simulated (lines) chip temperatures versus the in-plane heat fluxes dissipated in a bare hotspot (rectangles), a hotspot covered by a GBF (circles), a chip covered by a GBF with non-functionalized GO (up triangle) and a chip covered by a GBF with APTES FGO (down triangle). `Exp.' and `Sim.' stand for experiments and simulations, respectively. Inset: calibration relationship between the resistance *R* 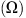 and the temperature *T* (°C) of the thermal evaluation chip. Scale bar on the chip, 100 μm. The error bars correspond to the s.d.'s from the measurements on five samples.

A GBF bonded to the FGO substrate through silane molecules is shown in Fig. 1a,b. To synthesize GBF and FGO experimentally (Fig. 1c), we first prepared a graphene oxide (GO) dispersion (see Experimental method). The FGO was obtained by functionalizing GO with a silane-based chemistry suitable for reactive oxide-forming surfaces including the basal plane of GO and SiO_2_. 3-Amino-propyltriethoxysilane (APTES) has three –Si–O– groups and one –NH_2_ end, as shown in Fig. 1a. Owing to the simple chemistry and unique multifunctional nature of APTES, it can easily bind two different substrates. In our case, the –Si–O end of APTES binds to the GO substrate. –Si–OC_2_H_5_ groups of APTES hydrolyse in water and form crosslink bonds with each other. The crosslinked Si–O structure acts as a strong bonding layer between the substrate and GBFs. On the other hand, the –NH_2_ end of APTES binds onto carboxyl groups on the functionalized graphene film. The FGO layer has a thickness of ∼5 nm. The graphene film was fabricated from chemically reduced GO and can recover relatively high in-plane thermal conductivity after thermal annealing^34^. The graphene film was then spin-coated^35^ with the FGO and the resulting bundle was transferred to a thermal evaluation device^7^, resulting in the formation of molecular bridges between the graphene surface and the device's SiO_2_ substrate. The thermal evaluation device was integrated with micro Pt-based heating resistors as the hotspot and temperature sensors^7^, as shown in Fig. 1d, acting as a simulation platform of an electronic component to demonstrate the heat-spreading capability of the supported graphene film.

### *In situ* temperature measurement with resistance thermometry

A direct current *I* was input into the circuit by applying an outer voltage *V* in Fig. 1e, and hence the generated power is calculated as *P*=*V* × *I*. Since the lateral dimension of the hotspot (A=400 × 400 μm^2^) is much larger than its thickness (260 nm), most of the heat is dissipated through the lateral direction of the hotspot. Hence the heat flux is defined as *Q*=*P*/*A*, and the direction is parallel to the substrate. The calibration relationship between the resistance *R* 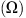 and the temperature *T* (°C) of the thermal evaluation chip is *R*(*T*)=0.21*T*+112. The temperature measurement uncertainty is *ε*=±0.5 °C. Figure 1e shows the temperature measured *in situ* at the hotspot and compares the thermal performance of the graphene film with and without the functionalization. With a constant heat flux of 1,300±2.3 W cm^−2^ at the Pt chip, the hotspot temperature decreased from 135±1.2 to 118±1.1 °C (Δ*T*=17±2.3 °C) with a GBF deposited on non-functionalized GO compared with the case of a bare chip. Such a remarkable temperature decrease is far beyond the measurement uncertainty Δ*T*>>*ε* (see Supplementary Fig. 1 and Supplementary Note 1 for an uncertainty analysis). Furthermore, with the same heat flux input, the hotspot temperature further decreased from 118±1.1 to 107±0.8 °C (Δ*T*=11±1.9 °C) thanks to the presence of the APTES functionalization. The heat-spreading performance is thus enhanced by ∼57% via the functionalization. We have implemented a finite-element model (Supplementary Figs 2–4, Supplementary Table 1 and Supplementary Note 2) of the heat-spreading device by taking the results of atomistic simulation as input parameters. As shown in Fig. 1e, the heat-spreading performance of the equivalent macroscopic finite-element model agrees reasonably well with the one measured by experiments.

### Thermal resistance measurement with photothermal reflectance

**Figure 2:**
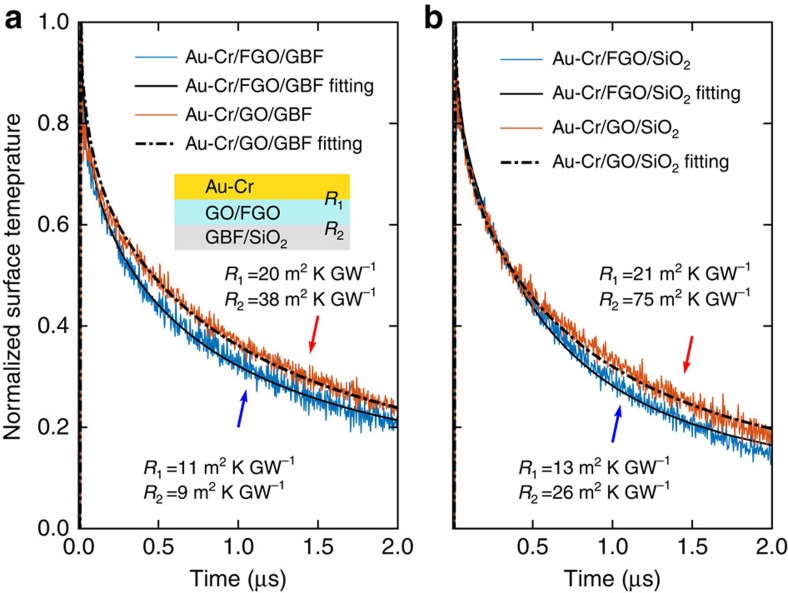
Normalized surface temperature of PPR measurements of the thermal interface resistance. (**a**) Au-Cr/FGO/GBF and Au-Cr/GO/GBF samples and (**b**) Au-Cr/FGO/SiO_2_ and Au-Cr/GO/SiO_2_ samples. Inset: sample geometry for the PPR measurement. *R*_1_ and *R*_2_ refer to the thermal interface resistance between the Au-Cr film and the (APTES functionalized) GO layer, and that between (functionalized) GO layer and GBF or SiO_2_. The thermal resistances are also reported in Table 1.

To further confirm the enhanced heat spreading assisted by molecular functionalization, we measured the interface thermal resistance by using the pulsed photothermal reflectance (PPR) method^36^,^37^. To enhance heat absorption, a gold layer was evaporated on the surface of the GO and FGO layers after drop coating. The sample was first excited by a Nd:YAG laser pulse. This caused a fast rise in the surface temperature followed by a relaxation. The change of surface temperature was monitored by a probe laser, which reflects off from the samples' surface. Since the relaxation time is governed by the thermal properties of the underlying layers and interfacial thermal resistance between the layers, by obtaining the temperature profile one can extract the thermal properties of the layers and interface thermal resistance between the layers through a heat conduction model. Four sets of samples were fabricated, as shown in Fig. 2 and the thermal resistance *R*_1_ between the Au-Cr film and the (functionalized) GO layer, and the resistance *R*_2_ between (functionalized) GO layer and GBF or SiO_2_ were measured. The experimental set-up and the procedure of thermal resistance extraction by fitting the photothermal response to the model (Supplementary Figs 5 and 6 and Supplementary Note 3). The normalized surface temperatures of the four sample sets are shown in Fig. 2 and the extracted thermal resistances are reported in Table 1. A fourfold reduction was achieved in the thermal resistance between GO and GBF from 3.8 × 10^−8^ to 0.9 × 10^−8^m^2^ K W^−1^. On the GO–SiO_2_ interface, the functionalization remarkably reduced the thermal resistance by a factor of almost three, from 7.5 × 10^−8^ to 2.6 × 10^−8^m^2^ K W^−1^. We also observed a better thermal coupling on the metal–dielectric interface between Au-Cr and GO due to the surface chemistry.

### Heat spreading through different functional molecules

**Figure 3:**
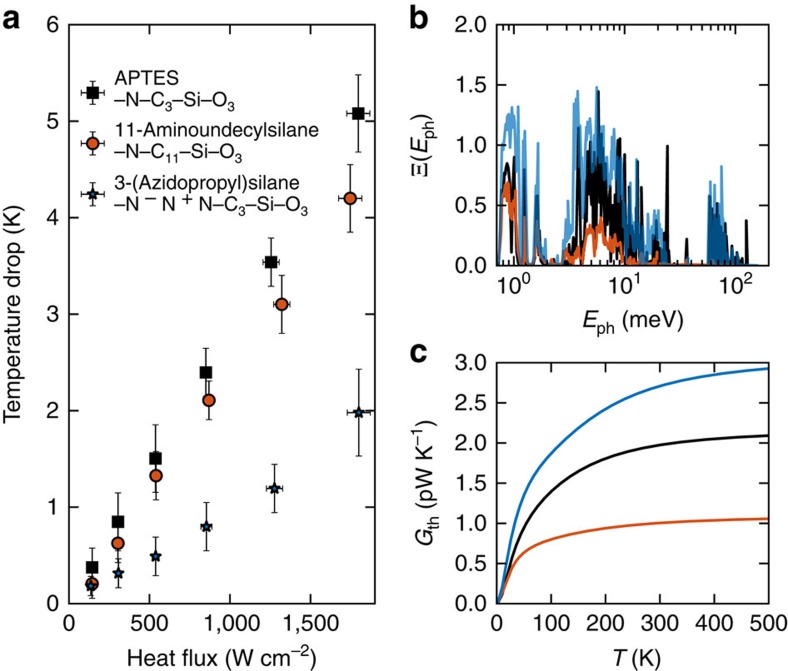
Heat-spreading performance of GBF on FGO with different functional agents. The molecules are APTES, 11-Aminoundecyltriethoxysilane and 3-(Azidopropyl)triethoxysilane. (**a**) Measured temperature drop of heat spreaders with different functionalization molecules compared with that with non-functionalized GO. The error bars correspond to the s.d.'s of measurements on five samples for each molecule type. (**b**) Phonon transmission function Ξ(*E*_ph_) for three different types of molecules used in the experiment. (**c**) Phonon thermal conductance through the molecules as a function of temperature. The colour code of the data curves in **b** and **c** is the same as in **a**.

To gain a deeper insight into the impact of molecular structure on the thermal transport along the molecules, we used APTES, 11-Aminoundecyltriethoxysilane and 3-(Azidopropyl)-triethoxysilane as different functional agent on the GO to evaluate their heat-spreading performance on the same thermal test platform. The same concentration of 0.1492, mol kg^−1^ was used for all three types of molecules. Figure 3 shows the temperature reduction of the hotspot covered by GBFs with FGO using three different molecules. The results show that the heat spreader of GO functionalized with APTES has the best cooling performance. To properly understand this difference, an exploration of the internal vibrational properties of the molecule is crucial^38^. We hence investigated how the differences in the phonon transmission impact the interfacial thermal transport. With this aim, we probed the phonon transmission Ξ(*E*_ph_) by atomistic Green's function to characterize the local heat conduction with and without the presence of the molecule (Supplementary Note 4). Ξ(*E*_ph_) enables a precise measurement of the atomic-scale molecule-graphene heat transport that the conventional models fail to provide. The phonon transmission functions through different molecules and the associated thermal conductances versus temperature are shown in Fig. 3b,c. The phonon transmission at low phonon energies across the 11-Aminoundecyltriethoxysilane molecule is comparable to that across the APTES molecule, whereas at high phonon energies (>4 meV), the phonon transmission is considerably suppressed. Such a distinct phonon transport behaviour is determined by the molecule chain length. By comparing the chemical structures, we can see that 11-Aminoundecyltriethoxysilane (–N–C_11_–Si–O_3_) has the same functional groups as APTES (–N–C_3_–Si–O_3_) but has a longer carbon backbone. Such a long chain length has a rather weak impact on the low-frequency phonons due to their very long wavelength but can strongly suppress the phonon transport at high frequencies. On the other hand, when comparing the phonon transmission through the junction of APTES and 3-(Azidopropyl)triethoxysilane (–N^−^N^+^N–C_3_–Si–O_3_), it is evident to identify a stronger transmission at all frequencies. By comparing the chemical structures in Fig. 3a, we can see that 3-(Azidopropyl)triethoxysilane has the same carbon backbone as APTES but has an azido group instead of an amino group. The azido group has strong interaction with the *sp*^2^-bonded carbon lattice of graphene to form three-membered heterocycle structures. Given the similar backbone structure, the phonon eigenmodes in the molecule have not been significantly altered, but the transmission is enhanced due to the stronger thermal coupling to the graphene reservoirs. However, the introduction of nitrogen atoms into the *sp*^2^ carbon structure can strongly scatter phonons by distorting the structure of the graphene sheet, which leads to a poor thermal performance of the heat spreader.

### Phonon transport analysis in APTES

**Figure 4:**
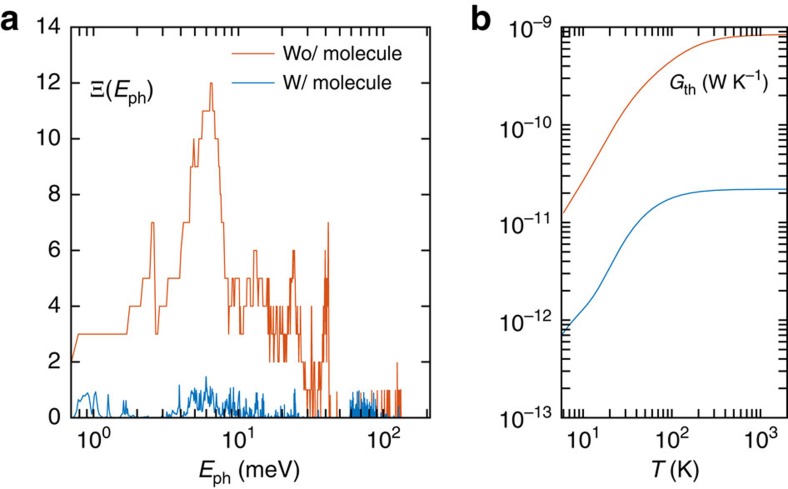
Phonon transport through the APTES molecule. (**a**) Phonon transmission Ξ(*E*_ph_) versus phonon energy 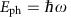 (red curve) between two adjacent graphene layers and (blue curve) through the APTES molecule bonding the two graphene layers. `w/' and `wo/' stand for with and without, respectively. (**b**) Thermal conductances *G*_th_ contributed by phonons versus temperature for the two cases.

We now investigate the detailed vibrational and electronic transport properties of the APTES molecule since it presents the best performance in heat spreading. We probed the phonon transmission Ξ(*E*_ph_) to characterize the local heat conduction with and without the presence of the APTES molecule. As shown in Fig. 4, the transmission function Ξ(*E*_ph_) for the two adjacent graphene layers without any molecule displays a clear stepwise structure that provides the number of phonon channels. Low-energy phonons (*E*_ph_≤10 meV) dominate heat conduction since the adjacent graphene flakes interact only through weak van der Waals (vdW) forces that inhibit the transmission of high-frequency phonons^39^. When the graphene layers are bridged by a amino-silane molecule, the high-frequency phonons act as the major contributors in the heat conductance *G*_ph_, creating more phonon channels through the covalent bond vibrations. This is in line with the transmission calculation of Segal *et al.*^40^ who observed a contribution to heat conduction by the higher-frequency phonons within the molecule coupled to the low-frequency phonons responsible for heat transport in the thermal reservoirs. The oscillations in the transmission spectrum may originate from phonon interferences within the alkane chain^41^,^42^,^43^. Fabry–Pérot-like interference effect occurs in the frequency region of *E*_ph_=20–100 meV, as was previously observed in an alkane SAM interface^41^. Such Fabry–Pérot-like interferences originate from the multiple reflected phonons interfering constructively within the alkane chain, as the local maxima in the transmission (Fig. 4) through the molecule can attain the same intensity of that through pristine graphene films at given frequencies. Although destructive quantum interference was believed unlikely to occur in a linear alkane chain^42^, we observe strong destructive interference patterns in the high-frequency range 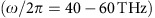, which may correspond to two-path destructive phonon interferences^43^,^44^. We also investigated the interlayer electron transport in the graphene and the effect of the silane intercalation in such a hybrid nanostructure through a *ab initio* calculation combined with Green's function (Supplementary Fig. 8 and Supplementary Note 5). The main heat carrier in this system is phonon, as the thermal conductance due to the electron contributes to the total thermal conductance by ∼4% at room temperature with functionalization and by ∼2% without molecules.

### Thermal conductance and conductivity calculations

To explore the effect of functional APTES molecules on the in-plane thermal conductance of the graphene film, we first perform molecular dynamics simulations to study a nanoscale molecular junction between two stacks of multilayer graphene nanoflakes. For a weak oxidation, the thermal resistance of the graphene film and its oxidized substrate on one hand, and on the other hand its thermal conductivity are very close to those with a non-oxidized graphene support. The dependence of thermal resistance and conductivity of graphene film to the oxidation rate of the GO substrate is reported in Supplementary Fig. 9 and Supplementary Note 6. We consider defect-free and isotopically pure graphene flakes of 10 × 10 nm^2^ thermalized at 300 K (Methods, Supplementary Fig. 10 and Supplementary Note 7). The FGO substrate has a 5-nm thickness as in the experiments. Conventional silica substrate results in a substantial decrease of the basal-plane thermal conductivity of graphene due to the non-conformality of the substrate–graphene interface^14^. To compare, the FGO substrate proposed herein minimize the perturbation of substrate on the morphology of graphene (Fig. 1c), thus maintaining its high thermal conductivity. Periodic boundary conditions were used in the in-plane directions so that the molecular dynamics system corresponds to two thin films connected through silane molecules with the number density *ρ*, defined as the molecule number per graphene unit area.

**Figure 5:**
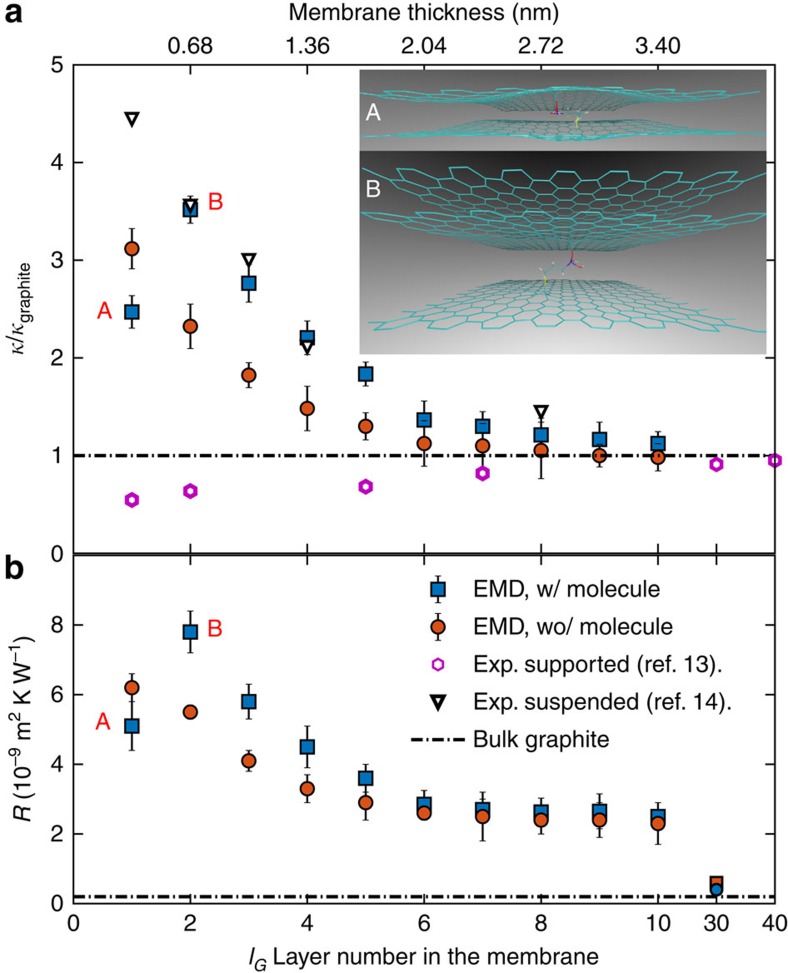
Thermal resistance and in-plane thermal conductivity of the GBF versus the graphene layer number. (**a**) Molecular dynamics simulation results of in-plane thermal conductivity 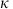 of the graphene film and (**b**) interfacial thermal resistance *R* between the FGO substrate and the graphene film versus the graphene layer number *l*_G_ in the film. The molecule density is *ρ*=0.081 nm^−2^. Cases with (red circles) and without (blue squares) the APTES molecule are compared. `w/' and `wo/' stand for with and without, respectively. The values of thermal conductivity are normalized to that of the single-layer graphene. Inset A illustrates a saddle-like surface generated by the APTES molecule for *l*_G_=1. Inset B shows that the saddle-like curvature disappears for *l*_G_≥1. The measurements of suspended and supported graphene are from refs 13, 14, respectively. EMD stand for equilibrium molecular dynamics. `Exp.' and `Sim.' stand for experiments and simulations, respectively. The simulated (respectively measured) thermal conductivity is normalized with respect to that of the simulated (respectively measured) graphite to allow a reasonable comparison. The error bar corresponds to the s.d. of the ensemble average on 50 independent trajectories.

To illustrate the intriguing role of the functionalizing molecule, the in-plane thermal conductivity 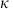 of the film and its interfacial thermal resistance *R* (Supplementary Fig. 10 and Supplementary Note 7) with the FGO substrate is plotted as a function of the graphene layer number *l*_G_ in the film in Fig. 5a,b, respectively. First, a supercell containing a single molecule is studied, which corresponds to *ρ*=0.081 nm^−2^. For *l*_G_≥2, the presence of the APTES molecule results in an unexpected increase both in the graphene film thermal conductivity 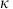 and in *R*. An overall decaying trend of the in-plane thermal conductivity 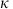 of the graphene film and its resistance *R* with the substrate versus the layer number *l*_G_ is observed until approaching the value of bulk graphite. This is due to the increased cross-plane coupling of the low-energy phonons. A similar decay was found both in experimental measurements of 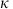 (ref. 13) of suspended graphene and in simulation-based estimation of *R* (ref. 45). Unlike silica-supported graphene^14^, the FLG on FGO support recovers the high thermal conductivity and follows the same decaying trend versus *l*_G_ as the suspended graphene^13^. Therefore, the proposed FGO substrate better conserves the high thermal conductivity of graphene compared with the silica substrate. Interestingly, for *l*_G_=1, the presence of the molecule reduces 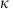, which goes against the case where no molecule interconnects the graphene film and the substrate. This breakdown of the thermal conductivity enhancement is due to a saddle-like surface generated around the molecule's chemical bonds of amino and silano groups connecting the graphene, with the bond centre as the saddle point, as shown in the inset A of Fig. 5a. The saddle-like surface strongly scatters all phonon modes, thus decreasing 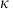 of the graphene film. Such a curved surface was found quite common in defective graphene^46^,^47^, resulting from the Jahn–Teller effect to lower the energy by geometrical distortion^48^. The thermal conductance of a single silane molecule is determined to be 82 pW K^−1^ through the molecular footprint (Supplementary Fig. 11 and Supplementary Note 8). Our simulated result is comparable to recent measurements of the thermal conductance of alkane thiols SAM at a silica–gold interface^38^.

It is clearly seen from Fig. 5a that the critical layer number for the in-plane thermal conductivity switch is *l*_c_=2. This critical number depends on the level of conformation distortion of the functionalized graphene sheet. Several factors can play a role (Supplementary Figs 12–14 and Supplementary Note 9): (i) molecule density: a diluted molecule distribution creates segregated ripples on the functionalized graphene sheet and even on the sheets further above thus interrupting the phonon mean free path. In this case more graphene sheets are required to recover the flat conformation, that is, *l*_c_>2. For a high molecule density, the ripples tend to merge with themselves, recovering the flat surface in an extended area. Such merging hence recovers the interrupted phonon MFP and alleviates the detrimental influence of the separate ripples on the basal-plane thermal conductivity of graphene. In this case the two layers of graphene with functionalization have higher basal-plane thermal conductivity compared with that without functionalization, that is, *l*_c_=2. (ii) Functional group of the molecule: when the functional agent forms a stronger bond with its substrate than that of the amino-silane molecule, more severe static graphene distortion occurs thus increasing the critical number *l*_c_. (iii) Substrate of the graphene film: a highly mismatched substrate yields remarkable surface distortion of the graphene sheets in such a way that two layers of graphene are not sufficient to recover the high basal-plane thermal conductivity.

### Functionalization effects on phonon lifetime of graphene

**Figure 6:**
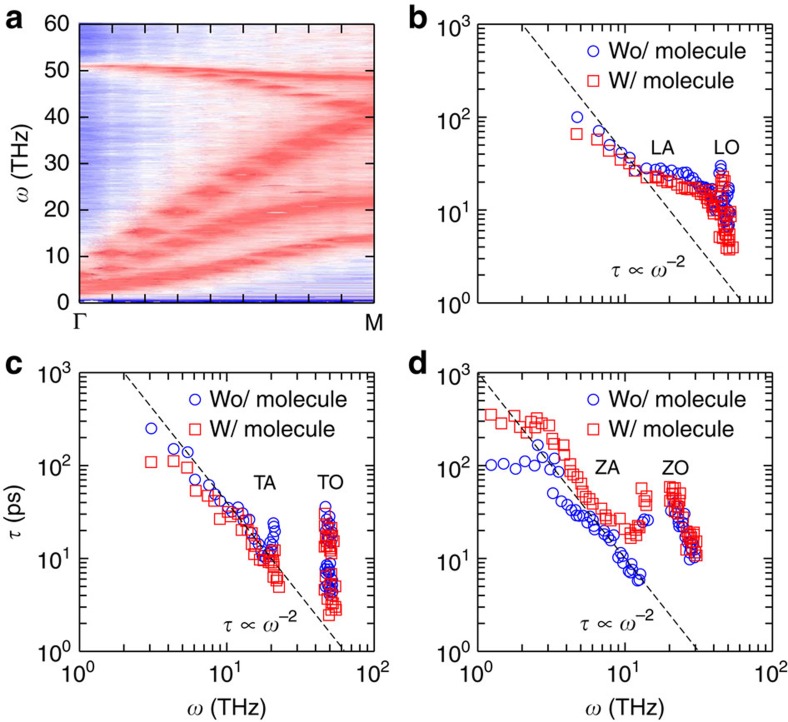
Phonon dispersion and lifetimes of the graphene film. (**a**) Phonon dispersion of the graphene film bonded to the APTES-FGO substrate from molecular dynamics simulations, for *ρ*=0.081 nm^−2^ and *l*_G_=2. (**b**–**d**) Mode-wise phonon relaxation time for longitudinal modes including longitudinal acoustic (LA) and optical (LO) branches, for transverse modes including transverse acoustic (TA) and optical (TO) branches, and for flexural modes including flexural acoustic (ZA) and optical (ZO) branches, respectively. `w/' and `wo/' stand for with and without, respectively. For low-frequency phonons, 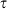 follows the 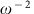 scaling rule of the Umklapp processes at low frequency and high-temperature limits.

We investigate the microscopic origin of the thermal conductivity 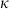 enhancement in the graphene film by probing the mode-wise phonon relaxation time (Supplementary Fig. 15 and Supplementary Note 10). The phonon relaxation time 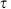 measures the temporal response of a perturbed phonon mode to relax back to equilibrium due to the net effect of different phonon-scattering mechanisms. 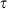 can be defined as^49^ 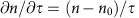 where *n* and *n*_0_ are the phonon occupation numbers out of and at thermal equilibrium. Under the single-mode-relaxation-time approximation, the thermal conductivity is given by 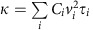 where *C*_*i*_ and *v*_*i*_ are the specific heat per volume unit and the group velocity of the *i*-th phonon mode. The phonon dispersion of the supported graphene film for *ρ*=0.081 nm^−2^ and *l*_G_=2, and the extracted relaxation time 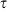 for all the phonon modes are shown in Fig. 6. By inserting the APTES molecule, the relaxation time of the acoustic flexural modes 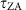 largely increase at low frequencies 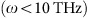, whereas the longitudinal and transverse modes undergo a slight decrease in 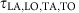. The notable increase in 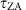 accounts for the enhancement in 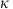 of the graphene film bonded to the substrate since the ZA modes contribute considerably to 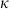 as much as 77% at 300 K (ref. 15). We attribute the increase in 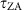 to the weakened coupling between the graphene film and the substrate, which is reflected as the increased thermal resistance *R* for *ρ*=0.081 nm^−2^ and *l*_G_≥2, as is shown in Fig. 5b. An approximate expression from the perturbation theory for the relaxation time due to phonon leakage towards the contact interface yields 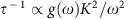, where 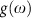 depends on the phonon density of states and *K* is the average vdW coupling constant between the graphene film and its substrate. A previous calculation^16^ shows that *K*_ZA_ largely depends on the substrate morphology. The presence of the molecule strongly decreases the film–substrate coupling *K*_ZA_, thus yielding a longer time 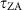. This demonstrates that the amino-silane molecules refrain the film–substrate phonon scattering, which in turn enhances in-plane heat conduction in the bonded graphene film. Note that ZA mode becomes ‘massive' as shown in Fig. 6a, that is, the ZA branch does not reach all the way to zero frequency but shifts to higher frequencies. The reduced inter-plane scattering compensates the reduction in the phonon group velocity at Brillouin zone centre, so that the in-plane thermal conductivity increases after all (Supplementary Fig. 16 and Supplementary Note 11).

**Figure 7:**
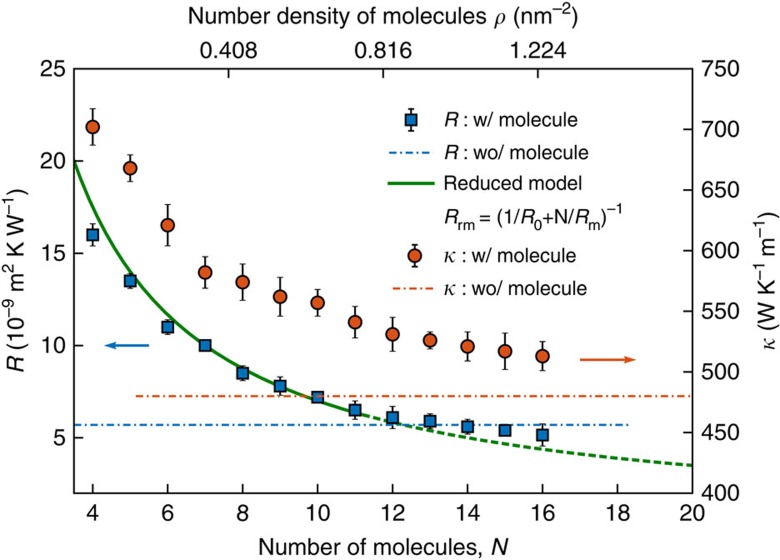
Thermal resistance and in-plane thermal conductivity of the graphene film versus the molecule density. Thermal conductivity 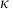 (red circles) of the graphene film and its thermal resistance *R* (blue squares) with the APTES functionalized substrate versus the equivalent molecule number density *ρ*. Green line: resistance *R*_rm_ predicted by a reduced model of parallel thermal resistors is to compare with the molecular dynamics (MD) results for small *ρ*. *R*_rm_=(1/*R*_0_+*N*/*R*_m_)^−1^, where *R*_0_ is the thermal resistance between two adjacent graphene layers and *R*_m_ is the additional resistance induced by a single molecule. The values of *R*_0_ and *R*_m_ are determined in MD simulations: *R*_0_=0.203 mm^2^ K W^−1^ and *R*_m_=0.069 mm^2^ K W^−1^. Red and blue dashed lines represent, respectively, 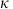 and *R* without the interconnecting molecule at the film–substrate interface. `w/' and `wo/' stand for with and without, respectively. The error bar corresponds to the s.d. of the ensemble average on 50 independent trajectories.

The cross-plane heat conduction between the top graphene films and the lower FGO layers is governed by the competing effects of (i) the intercalation of the molecules that tends to weaken the interlayer coupling of graphene therefore increasing the cross-plane thermal resistance and (ii) the additional heat channels introduced by the molecules covalently bonding the graphene sheets that tend to facilitate the cross-plane thermal coupling. We investigated the in-plane thermal conductivity 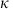 of the graphene film and its thermal resistance *R* with the functionalized substrate versus the equivalent APTES molecule number density *ρ* in Fig. 7. At a low molecule density, the molecule population is too small in such a way that the additional thermal phonon transport through the molecules cannot compensate the weakened cross-plane thermal coupling through the interlayer vdW interactions due to the intercalation effect by the molecules (also see Supplementary Fig. 17 and Supplementary Note 12 for a detailed analysis on the *κ*–*ρ* relation at low molecular densities). Therefore, the net effect is an increase in the cross-plane thermal resistance between the top graphene films and the lower GO layers compared to the non-functionalization case. In the limit of small number density, we consider the molecules as independent heat conductors connecting the film and the substrate. *R* agrees well with a reduced model of parallel thermal resistors. Thus, *R* decreases with the number of molecules. 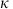 also decreases with the molecule number since the molecules increase the cross-plane phonon scattering in the graphene film. For larger *ρ*, the interactions among the molecules gain importance and *R* starts to deviate from the prediction of the parallel-thermal-resistor model, as shown in Fig. 7. The molecule population is large enough so that the additional thermal phonon transport through the molecules becomes very strong and compensates the weakened cross-plane thermal coupling through the interlayer interactions due to the intercalation effect by the molecules. Therefore, the net effect is that the cross-plane thermal resistance between the top graphene films and the lower FGO layers falls lower than that of the case without functionalization in the GO. For large *ρ*, the thermal resistance *R* is lower than that without molecules. However 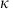 remains enhanced by a factor of ∼15% compared with its value for the non-functionalized graphene substrate. The molecule density *ρ* effectively tunes the thermal conductivity of the supported graphene.

## Discussion

The correlation between the in- and cross-plane thermal transport of 2D materials has been recently studied from both theoretical^15^,^17^ and experimental^13^,^14^,^16^ points of view. Among them, Ghosh *et al.*^13^ studied the dimensional crossover from a 2D graphene to a 3D graphite system. The in-plane thermal conductivity decreased with the increase of the layer number in the FLG. Wei *et al.*^17^ used a 2D material model to show a negative correlation between the in-plane thermal conductivity and the cross-plane interaction. These studies have considered different mechanisms of the correlation, yet no general conclusion is applicable to any quasi-2D system. We show that the functionalization molecules mediate the cross-plane phonon scattering and in turn effectively control the in-plane thermal transport in the graphene-based heat spreader.

Our work tackles the key technological challenge of efficient thermal management in the industry of the next-generation integrated circuit. The excessive heat from the hotspots accelerates the failure rate and slows down the operating speed of microelectronic devices^50^,^51^. Extremely high power density beyond 1,000 W cm^−2^ can be observed in microprocessors^51^. Coolers are most commonly based on metallic materials due to their relatively high thermal conductivities, for example, 200–400 W m^−1^ K^−1^ for Al and Cu, and the low cost. However, for these technologically important metals, the thermal conductivity of a 100-nm-thick film constitutes only ∼20% of the thermal conductivity of the bulk^52^,^53^. On the other hand, GBF used in the present work can have an in-plane thermal conductivity as high as 1,600 W m^−1^ K^−1^ (ref. 54), which is more than two orders of magnitude higher than the ones of the metal thin films. Such a superior thermal conductivity significantly facilitates the heat conduction when GBFs are used as heat spreaders together with a better cross-plane thermal coupling with FGO. The APTES functionalization yields an additional temperature decrease Δ*T* of the hotspot of 11 °C compared with the case of GBF with non-functionalized GO (Δ*T*=17 °C), resulting in a large total temperature decrease of 28 °C when operating at 1,300 W cm^−2^. In comparison, the highest Δ*T* achieved so far at a similar high heat flux of 1,250 W cm^−2^ is 19 °C by using a miniaturized thermoelectric cooler^51^. The contribution of functionalization to the overall Δ*T* is robust especially at higher heat flux beyond 1,000 W cm^−2^, as shown in Fig. 1e. Therefore, we propose a significant package-level solution for the thermal management of hotspots in high-power electronics at the micro- and nanometre length scale.

## Methods

### Experimental method

(i) Sample synthesis: graphite (Sigma, 4 g); H_2_SO_4_ (92 ml, 98%); NaNO_3_ (2 g); and KMnO_4_ (12 g) were used to prepare GO dispersion by following Hummers' method^55^. The obtained GO dispersion was reduced by L-ascorbic acid, and polyvinyl alcohol was also added for better suspension. The GBF was prepared via vacuum filtration with polycarbonate filter paper with a pore size of 3 μm. The film thickness was controlled by the filtration volume and the graphene concentration in the suspension. After dissolving the filter paper in pure acetone, a freestanding GBF was obtained. The thickness of the GBF was measured as ∼20 μm. Raman spectroscopy data of the GBF before and after the spin-coating with FGO are shown in Supplementary Fig. 18. (ii) Functionalization: GO powder (20 mg) and dicyclohexylcarbodiimide (5 mg) were mixed with APTES (30 ml) by ultrasonication for 2 h to produce a homogeneous suspension. Then the suspension was heated up to 100 °C for 3 h, with continuous stirring to realize the functionalization. Fourier transform infrared spectroscopy data provide evidence for the functionalization (Supplementary Fig. 19). (iii) Transfer process: the GBF was first transferred onto a thermal release tape, and then spin-coated^35^ with the FGO layer at 4,000 r.p.m. for 2 min onto the film. The thermal release tape was removed by heating the device.

### Simulation set-up

Classical molecular dynamics simulations were performed using LAMMPS^56^. Adaptive intermolecular reactive empirical bond order potential^57^ was used to simulate the graphene's C–C interactions. The intramolecular forces are taken into account through the ReaxFF potential^58^, which uses distance-dependent bond-order functions to represent the contributions of chemical bonding to the potential energy. Periodic boundary conditions are applied in the in-plane directions and free boundary condition in the cross-plane direction of the graphene system. First, each supercell was relaxed at the simulation temperature to achieve zero in-plane stress. Then the systems were thermalized by using a Langevin heat bath. The system reached thermal equilibrium after 2 ns computed in the microcanonical ensemble. Temperature and heat flux were sampled in the microcanonical ensemble in the in the following 1 ns. Autocorrelation functions for the resistance and the thermal conductivity were calculated over this latter duration.

### Thermal conductance

Thermal conductance 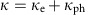, where 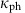 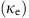 is the thermal conductance due to the phonons (electrons). From the phonon transmission Ξ(*E*_ph_) the thermal conductance due to the phonon could be calculated as 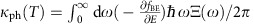, where 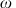 is the frequency, *T* refers to the mean temperature of the system, *f*_BE_ is the Bose–Einstein phonon statistics, and *ħ* represents the reduced Plancks constant. The thermal conductance due to the electrons^59^ could be calculated from the electron transmission coefficient *T*_el_ as 
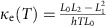
, where 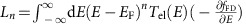 in which *f*_FD_ is the Fermi–Dirac electron statistics.

## Additional information

**How to cite this article:** Han, H. *et al.* Functionalization mediates heat transport in graphene nanoflakes. *Nat. Commun.* 7:11281 doi: 10.1038/ncomms11281 (2016).

## Supplementary Material

Supplementary Information Supplementary Figures 1-19, Supplementary Table 1, Supplementary Notes 1-14 and Supplementary References.

## Figures and Tables

**Table 1:** Au/(F-)GO and (F-)GO/X interface thermal resistances.

**Interface**	***R***_**1**_ **(m**^**2**^ **K** **W**^**−1**^)	***R***_**2**_ **(m**^**2**^ **K** **W**^**−1**^)
Au-Cr/GO/GBF	2.0E-8	3.8E-8
Au-Cr/FGO/GBF	1.1E-8	0.9E-8
Au-Cr/GO/SiO_2_	2.1E-8	7.5E-8
Au-Cr/FGO/SiO_2_	1.3E-8	2.6E-8

X=GBF or SiO_2_. The fitting uncertainty is 20% (Supplementary Fig. 7).
