# Supplementary material for: Functionalization mediates heat transport in graphene nanoflakes
Source: Nat Commun. 2016 Apr 29;7:11281. doi: 10.1038/ncomms11281 (PMC4855536; doi:10.1038/ncomms11281)
Supplement: Supplementary Information — Supplementary Figures 1-19, Supplementary Table 1, Supplementary Notes 1-14 and Supplementary References. [file ncomms11281-s1.pdf]

SUPPLEMENTARY FIGURES

Supplementary Figure 1

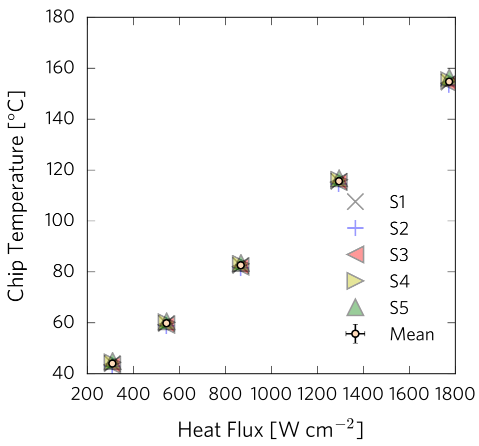

(a)

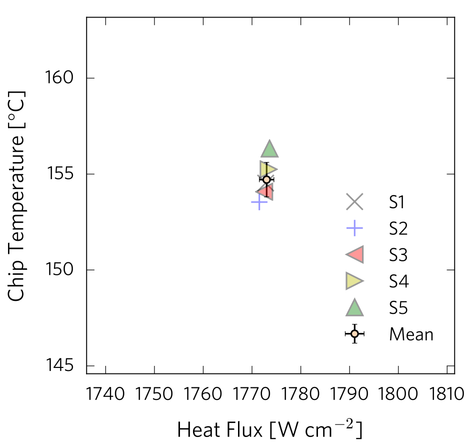

(b)

Supplementary Figure 1: (a) Example of measured temperatures versus the heat fluxes in the device for a sample set. Each sample set containing five samples S1-S5 are measured. (b) A zoom-in view of five measurements carried out at a heat flux of 1773 W cm^{-2}.

Supplementary Figure 2

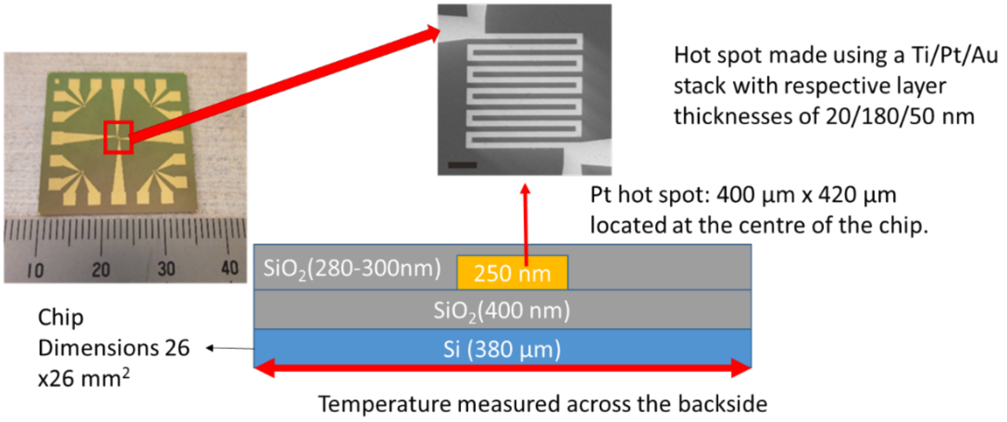

Supplementary Figure 2: Schematic of test chip FE validation model, in comparison to the test chip fabricated for the experimental work.

Supplementary Figure 3

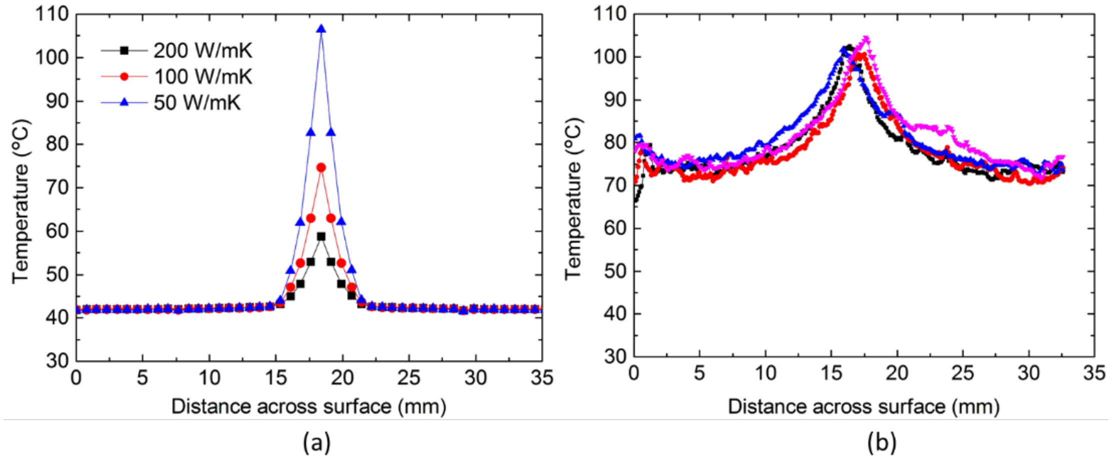

Supplementary Figure 3: Backside temperature measurements taken by (a) FE simulation of hot spot test chip for different thermal conductivities of Si and (b) line scans from an experimental test chip measured with an IR camera.

Supplementary Figure 4

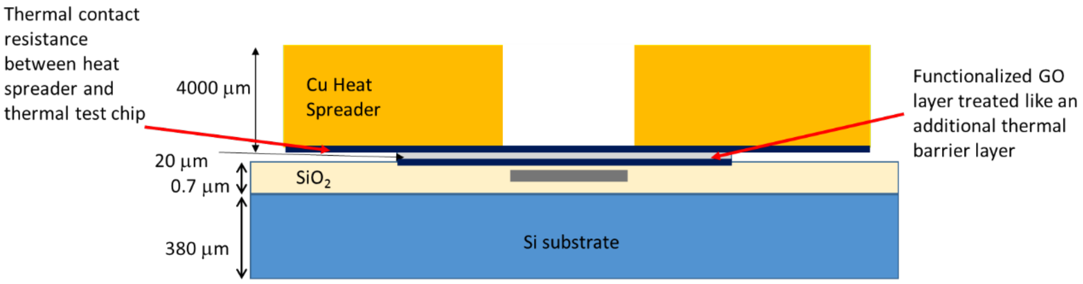

Supplementary Figure 4: Schematic of GBF heat spreader experiment inputted into ANSYS FE simulation software. The Cu heat sink is based on CAD drawing of the heat sink used experimentally.

Supplementary Figure 5

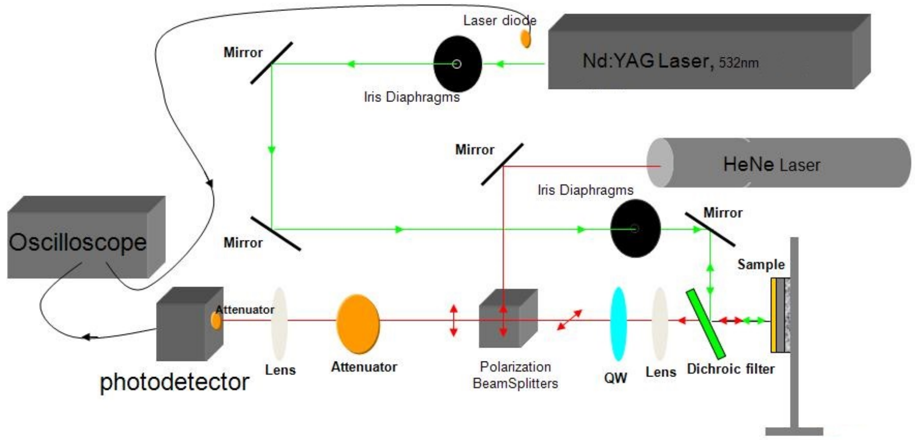

Supplementary Figure 5: Schematic diagram of PPR experiment.

Supplementary Figure 6

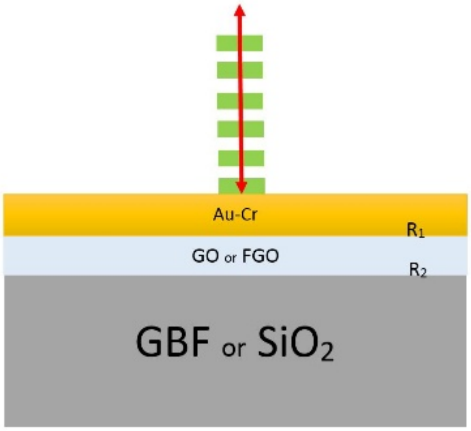

Supplementary Figure 6: Sample geometry used for the PPR measurement.

Supplementary Figure 7

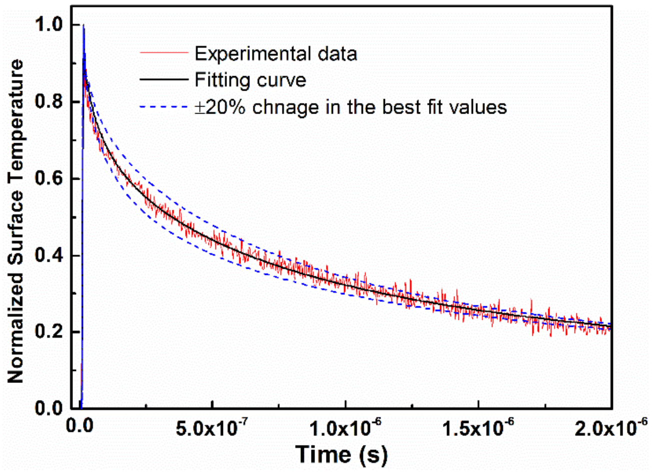

Supplementary Figure 7: Error in fitting the normalized surface temperature.

Supplementary Figure 8

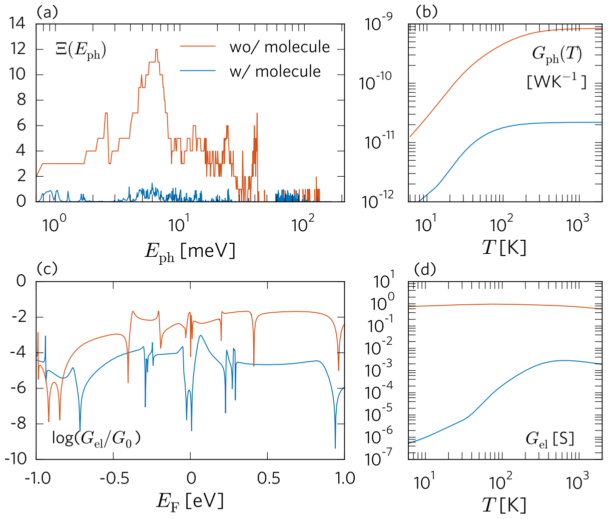

Supplementary Figure 8: Phonon transport through the APTES molecule. (a) Phonon transmission $\Xi(E_{ph})$ versus phonon energy $E_{ph} = \hbar\omega$ (red curve) between two adjacent graphene layers and (blue curve) through the APTES molecule bonding the two graphene layers. (b) Thermal conductances $G_{th} = G_{ph} + G_e$ contributed by phonons G_{ph} and by electrons G_e (dashed lines) versus temperature for the two cases. (c) Electron transmission $T_{el} = G_{el}/G_0$ versus Fermi energy E_F between two adjacent graphene layers and (blue curve) through the APTES molecule bonding the two graphene layers. (d) Electronic thermal conductances $\kappa_{el}(T)$ versus temperature for the two cases.

Supplementary Figure 9

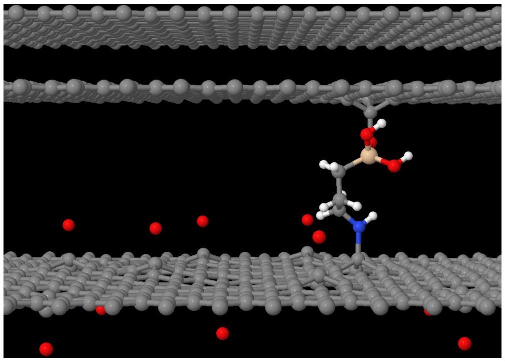

(a)

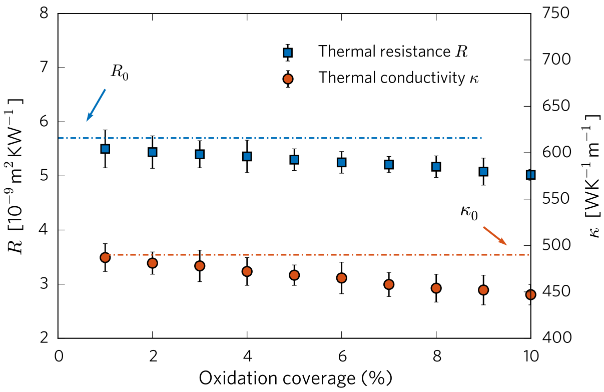

(b)

Supplementary Figure 9: Effect of oxidation on the thermal conductivity of graphene and thermal resistance. (a) Atomic presentation of the graphene film supported on the functionalized graphene oxide. The oxidation coverage is defined as the oxygen atom number versus the carbon atom number. The oxidation coverage as shown is 2%. (b) Thermal conductivity κ (red circles) of the graphene film and its thermal resistance R (blue squares) with the functionalized graphene oxide support versus the oxidation coverage. R_0 and κ_0 refer to the graphene film-support thermal resistance and basal-plane thermal conductivity of the graphene film without oxidation of the graphene support.

Supplementary Figure 10

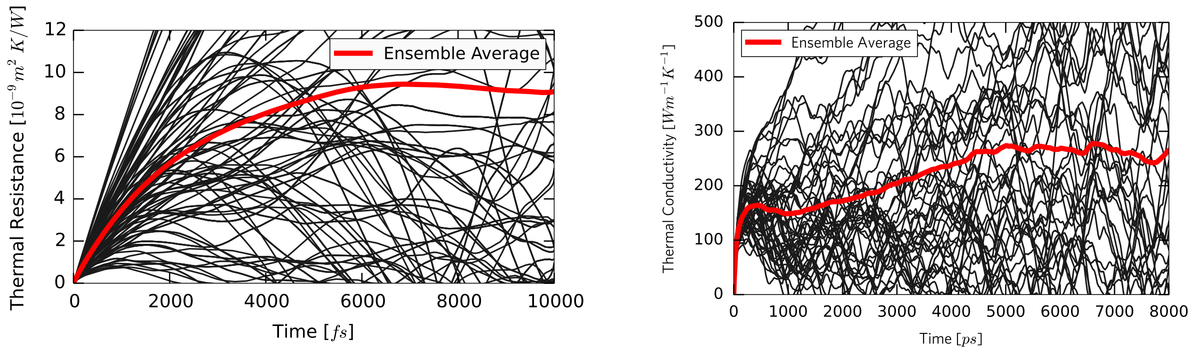

(a) (b)

Supplementary Figure 10: Ensemble average of trajectories to obtain thermal conductivity and thermal resistance. (a) Thermal resistance proportional to the time integrals of the normalized autocorrelation functions of the temperature difference from Eq. (8). (b) Thermal conductivity proportional to the time integrals of the heat flux autocorrelation functions from Eq. (9). Black curves are the running integral of a single trajectory and the red curve refers to the ensemble average of different trajectories.

Supplemental Figure 11

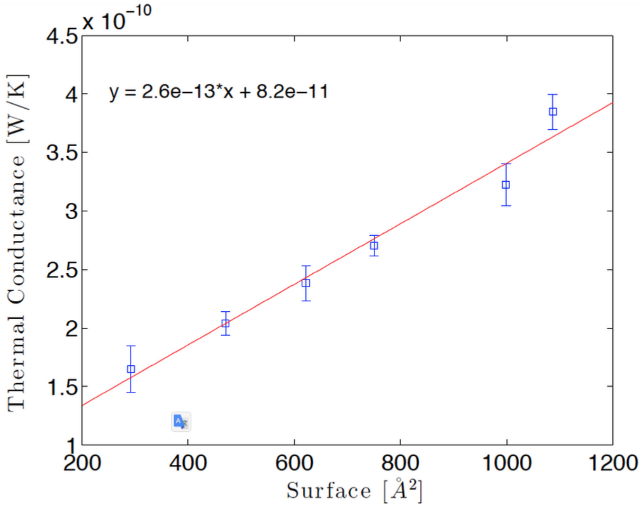

Supplementary Figure 11: Total thermal conductances for graphene systems of different contact area..

Supplemental Figure 12

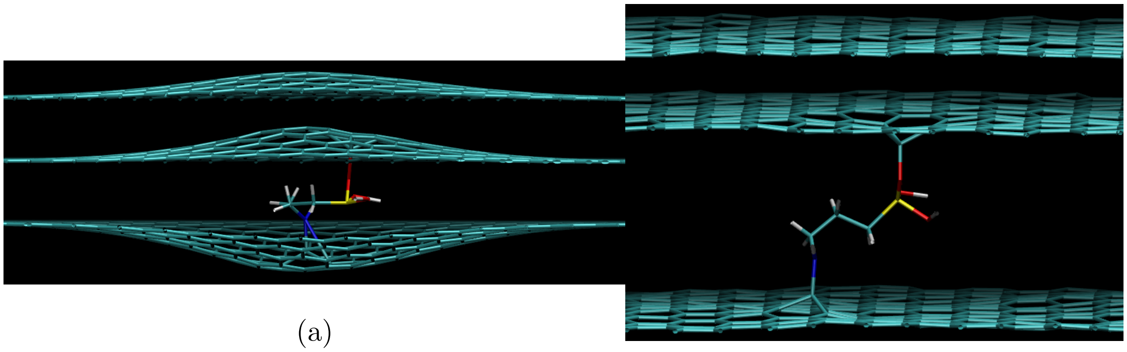

(a)

(b)

Supplementary Figure 12: Conformation of graphene supported on a functionalized graphene layer (a) for a low molecule density, $\rho = 0.05$ nm^{-2} and (b) for a high molecule density, $\rho = 0.41$ nm^{-2}.

Supplemental Figure 13

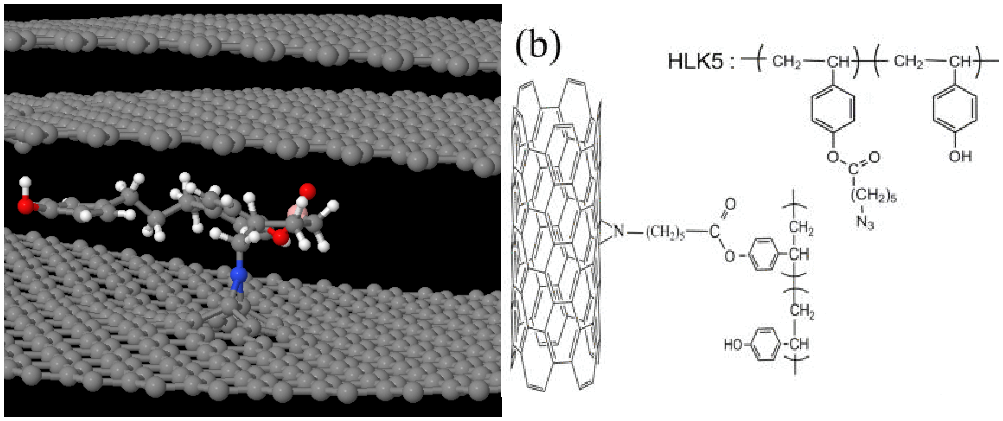

(a) (b)

Supplementary Figure 13: (a) HLK-5 covalently bonded to graphene. (b) Chemical representation of HLK-5 bonded to a carbon nanotube, extracted from Ref. [19] of the manuscript.

Supplemental Figure 14

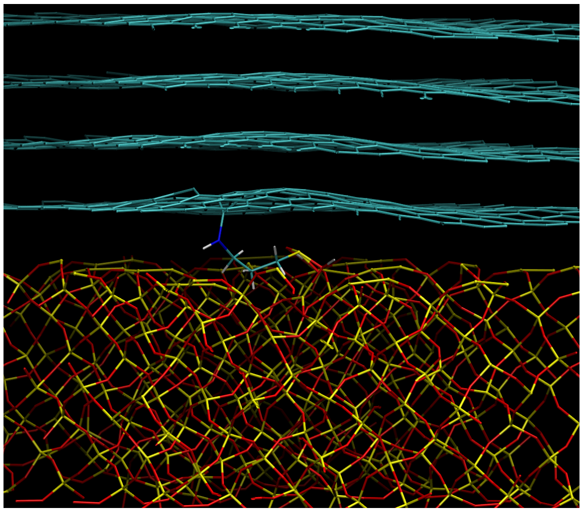

Supplementary Figure 14: Graphene film bonded through the amino-silane molecule to a silica substrate.

Supplementary Figure 15

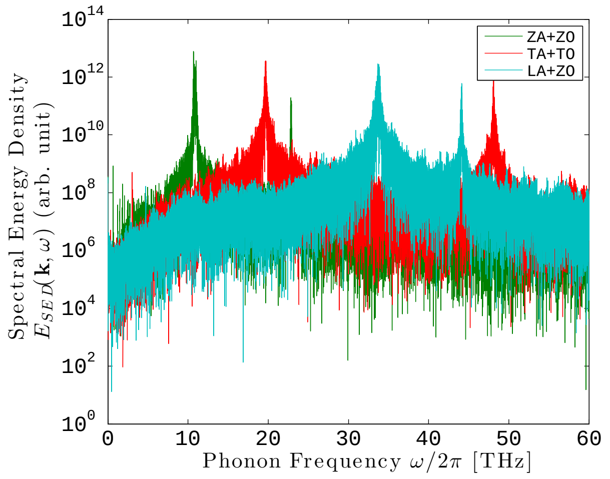

Supplementary Figure 15: Semilogarithmic plot of the spectral energy density function $E_{\mathrm{SED}}(\mathbf{k},\omega)$ along the Γ-K direction with $\mathbf{k} = \frac{4}{5}\frac{2\pi}{a_0}[100]$ of a supported graphene sheet. Different colors stand for the corresponding phonon branches.

Supplementary Figure 16

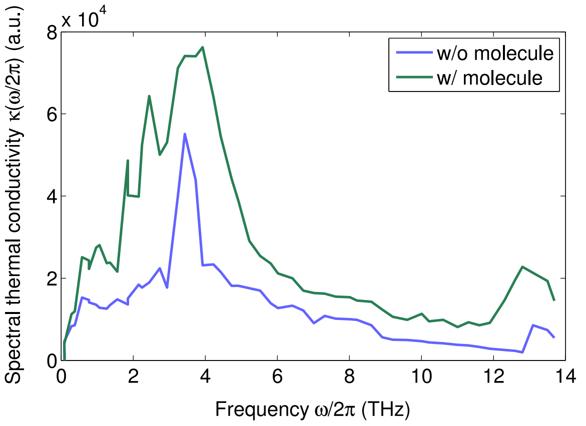

Supplementary Figure 16: Calibration results of the thermal evaluation chip. (Inset) Optical microscopic image of the Pt micro-heater, acting as the hot spot hotspot at the center of a thermal test platform.

Supplementary Figure 17

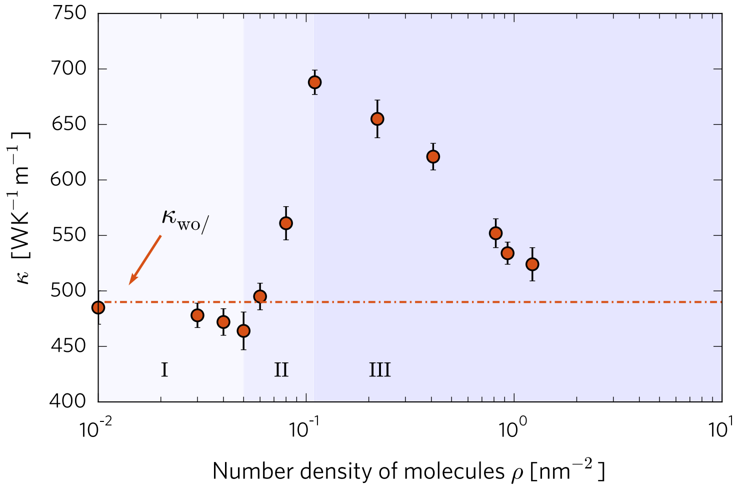

Supplementary Figure 17: Thermal conductivity κ (red circles) of the graphene film with the functionalized graphene substrate versus the molecule number density ρ. Red dashed lines represents κ wo/ without the interconnecting molecule at the film - substrate interface. The data are extracted from molecular dynamics simulations on a graphene structure with a basal-plane area of 10 nm $\times$ 10 nm. T. Meier et al. used a self-assembled monolayer with a number density ρ of 4.67 nm^{-2} [15].

Supplementary Figure 18

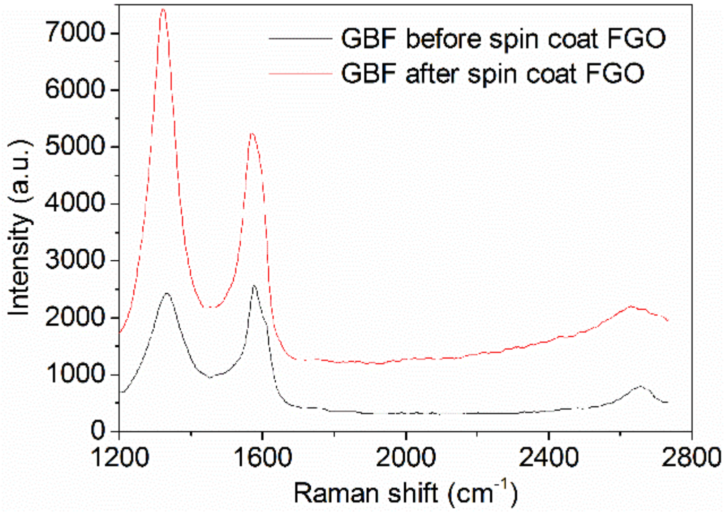

Supplementary Figure 18: Raman spectra of GBF before and after spin coating with FGO.

Supplementary Figure 19

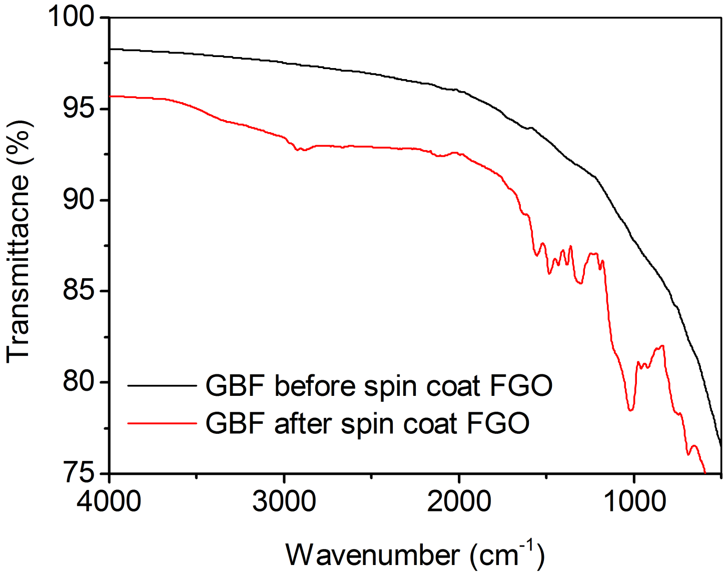

Supplementary Figure 19: FTIR spectra of GBF before and after spin coating with FGO.

SUPPLEMENTARY TABLE 1

Material	κ_{in} (W/m/K)	κ_{in} (W/m/K)	ρ (g/cm^3)	C_p (J/Kg/K)
Si	80[a]	80[b]	2.3	700
Graphene-based film	1640	-	1.2	760
Cu	401	401	8.3	385
SiO_2	1.5	1.5	2.2	745
Pt hot spot	72	72	21.5	133

[a] based on FLIR measurement

[b] based on FLIR measurement

Supplementary Table I: Key material properties used in the FE model.

SUPPLEMENTARY NOTE 1. UNCERTAINTY ANALYSIS ON THE TEMPERATURE AND HEAT FLUX MEASUREMENT

Two sets of samples of heat spreaders each containing five samples prepared under the same experimental condition were fabricated, composed of a graphene-based film coated on

- graphene oxide,
- functionalized graphene oxide.

In Fig. 1, we show a typical example of the measurement on the five samples of the control sample set (without functionalization).

The uncertainty of the heat flux measurement is due to the slight fluctuations in the direct current power supply (Agilent E3633A), especially at a high input power, but it is maintained at a low level of 1%. Standard calibration was carried out using a resistance temperature detector (RTD) (LAB FACILITY PT100 XF-316-FAR) at various temperatures in a furnace. The four-point probe methodology was implemented to measure the electric resistance and the applied power of the Pt temperature sensor. The error from the RTD measurement is as small as $\pm 0.5^{\circ}$C. The errorbar represents the standard deviation of the temperatures and the heat fluxes from the average value of the five samples. As can be seen from Fig. 1 above and Fig.1(e) of the manuscript, the additional temperature drop of 11°C due the functionalization in the heat spreader is much more significant compared to the uncertainty of 1.9^{o}C in the temperature measurements. Therefore the enhanced heat spreading performance due to the functionalization is confirmed with the uncertainty taken into account properly.

SUPPLEMENTARY NOTE 2. FINITE-ELEMENT MODELING OF THE THERMAL EVALUATION PLATFORM

Here we give details and discussion about the FE simulation of the enhanced heat spreading due to the functionalization. Based on experimental geometry, a transient thermal finite element (FE) model was created in ANSYS Workbench 16.0. In order to accurately simulate the experimental structure, a fully 3D experimental structure was created based upon CAD drawings of the Cu heat sink and test chip. The wiring of the test chip was too complicated

to be included in the simulation, so was simplified as a rectangular hotspot with dimensions of $400 \times 420\ \mu m^2$. The geometry of the test chip simulation is shown in Supplementary Figure 2. To simplify the hotspot structure further in the model, the entire metallic stack was assumed to be all platinum.

A heat flux of 1500 $Wm^{-1}K^{-1}$ was then applied to the test chip both experimentally and in the FE simulation, by running a transient thermal simulation for a time period of 1000 seconds. In the FE model, convection boundary conditions of 20 $Wm^{-1}K^{-1}$ were applied to the outer surfaces of the test chip so that it reached equilibrium with its surroundings. The ambient temperature of the surroundings was set to 22°C. Experimentally the temperature distribution of the test chip was measured from its backside using a FLIR camera. These results were compared and used to validate the test chip model. As the results in Supplementary Figure 3 show, the heat produced by the wiring in the test chip means the experimentally measured temperature at the edge of the chip is higher than in the simulation.

The peak experimental hotspot temperature corresponds to a thermal conductivity of 80 $Wm^{-1}K^{-1}$ for the Si test chip in the model. This is lower than the accepted thermal conductivity value of Si, but can be readily explained by the mesh convergence. Since solving the model required almost all available computational power, it was not possible to refine the mesh as much as wanted and ideally more layers of elements in the vertical direction would have been used if available. However, since the objective of this validation was to obtain a FE test chip with a similar temperature profile to the experimentally measured chip in order to accurately model the effect of the GBF, the validation was sufficient.

The GBF and Cu heat sink were then added to the simulation. The heat sink and GBF both have identical geometries as the experimental work. A schematic of the structure is contained in Supplementary Figure 4. The graphene oxide interlayer has been treated as one large thermal resistance as it only has a layer thickness of 5 nm, which is too thin to be reliably simulated. This thermal resistance was determined by the PPR measurements.

The PPR thermal resistance measurements were used to determine the effective resistance of both the GO and FGO interlayers using the following formula,

$$R_{tot} = R_1 + R_2 + d/K \tag{1}$$

where R_1 is the resistance between the GBF and GO interlayer, and R_2 is the resistance between the GO interlayer and SiO_2. R_{tot} denotes the total resistance of the GO/FGO

interlayer and the two thermal resistances on each side. Since both the GO and FGO interlayers have a thickness of about 5 nm, the d/K term becomes negligible. Therefore, the total thermal resistances of the GO and FGO layers and their interfaces are 1.13E-7 and 3.48E-8 m^2KW^{-1} respectively. Finally the thermal contact resistance between the heat spreader and the test chip with the GBF has been estimated at 1E-5 m^2KW^{-1} as it just sits on the rest of the sample and this gave a result close to experiment for the bare chip. These values were used in Figure 1c of the manuscript, where the model closely follows experimental trends. The simulation also predicts that the functionalization process offer a small additional decrease in hotspot temperature when compared to a GO interlayer without functionalization, agreeing with experimental findings.

The in-plane thermal conductivity κ in and cross-plane thermal conductivity κ_{cross}, densities ρ and specific heat C_p of the materials used in the model are contained in Supplementary Table I.

The chip emits a constant power Q as the heat source. A constant temperature of $T_0 0 = 273$ K was applied to the bottom boundary of the silicon wafer. Thermal insulation condition $-\mathbf{n} \cdot (-\kappa \nabla T) = 0$ was applied to the other outter boundaries of the setup. The heat dissipation process was simulated in the stationary regime by solving the well-known heat equation,

$$\rho C_p \mathbf{u} \cdot \nabla T = \nabla \cdot (\kappa \nabla T) + Q \tag{2}$$

where ρ is the mass density, C_p is the specific heat, $\mathbf{u}$ is the velocity vector and Q is the thermal power generated in the chip.

SUPPLEMENTARY NOTE 3. THERMAL INTERFACE RESISTANCE MEASUREMENTS

Pulsed Photothermal Reflectance (PPR) method is used to measure the thermal interface resistances. Supplementary Figure 5 shows the setup of the pulsed photothermal reflection measurement used in this study. To enhance the heat absorption, a gold layer is evaporated on the surface of the GO and FGO layers after dip coating. The sample is first excited by a Nd:YAG laser pulse. This causes a fast rise in the surface temperature and then followed by a relaxation. The change of surface temperature is monitored by a probe laser which

reflects off from the samples' surface. Since the relaxation time is governed by the thermal properties of the underlying layers and interfacial thermal resistance between the layers, by obtaining the temperature profile one can extract the thermal properties of the layers and thermal interface resistance between the layers through using a heat conduction model.

Fig.2(a) of the manuscript shows the normalized surface temperature obtained from the FGO/GO deposited on GBF. To extract the thermal interface resistance between the FGO/GO and GBF, the temperature excursion profile is fitted to a solution of the heat diffusion equation for three-layer stack model using thermal interface resistance between the layers as a fitting parameter [1]. The obtained thermal interface resistance between GO and GBF was 3.8E-8 m^2KW^{-1} and decreases to 0.9E-8 m^2KW^{-1} for the thermal interface resistance between FGO and GBF. Moreover, the thermal interface resistance of GO/SiO_2 and FGO/SiO_2 were also investigated. The obtained thermal interface resistance between GO/SiO_2 substrate was 7.5E-8 m^2KW^{-1} and it decreased to 2.6E-8 m^2KW^{-1} for that between FGO and the SiO_2 substrate, as shown in Fig.2(b). The results show that the thermal interface resistance for the FGO/GBF and FGO/SiO_2 interfaces are lower than the thermal interface resistance between the GO/GBF and GO/SiO_2 interfaces.

The samples geometry for the PPR measurement in this study is described in Supplementary Figure 6. In order to extract the thermal interface resistance between the layers, the photothermal response profiles are fitted with the analytical model of a one-dimensional heat equation in a multilayer structure. The fitting parameters are the thermal interface resistance between gold layer and GO/FGO layers R_1 , and thermal interface resistance between GO/FGO layers and GBF substrates R_2 . The obtained thermal interface resistances are listed in Table I of the manuscript and the error for thermal interface resistance measurements are about 20%.

SUPPLEMENTARY NOTE 4. THERMAL CONDUCTANCE AND PHONON TRANSMISSION USING ATOMISTIC GREEN'S FUNCTION

In the scattering theory, the system contains three coupled subsystems: two semi-infinite leads connected through the scattering region. The heat flux flowing in along the system axis writes

$$J = \int_{BZ} \hbar\omega_{\mathbf{k}} v_{g,\mathrm{k_z}} (n_L - n_R) t_{\mathbf{k}} \frac{\mathrm{d}^3\mathbf{k}}{(2\pi)^3} \quad (3)$$

where $\hbar\omega_{\mathbf{k}}$ is the energy quantum of the phonon mode $\mathbf{k}$, $v_{g,\mathrm{k_z}}$ is the phonon group velocity of the phonon mode $\mathrm{k_z}$, $n_{L,R}$ is the phonon number on the left and right reservoir following the Bose-Einstein distribution $n = \left(e^{\frac{\hbar\omega}{k_B T}} - 1\right)^{-1}$, $t_{\mathbf{k}}$ is the normalized transmission probability of the phonon mode $\mathbf{k}$ and $t_{\mathbf{k}} \in [\,0, 1\,]$. The integration goes through all the phonon modes in the irreducible Brillouin Zone (BZ). In the linear regime, the phonon population undergoes small perturbations and thus the thermal conductance writes

$$G = J/\Delta T = \int_{BZ} \hbar\omega_{\mathbf{k}} v_{g,\mathrm{k_z}} \frac{\partial n}{\partial T} t_{\mathbf{k}} \frac{\mathrm{d}^3\mathbf{k}}{(2\pi)^3} \tag{4}$$

We note that $\mathrm{d}^3\mathbf{k} = \mathrm{d}k_x \mathrm{d}k_y \mathrm{d}k_z$ and $v_{g,\mathrm{k_z}} \mathrm{d}k_z = \partial\omega/\partial k_z \mathrm{d}k_z = \mathrm{d}\omega$. The Eq. (4) reduces to

$$G = J/\Delta T = \int_{BZ} \hbar\omega \frac{\partial}{\partial T} \left(e^{\frac{\hbar\omega}{k_B T}} - 1\right)^{-1} [t_\omega \mathrm{d}k_x \mathrm{d}k_y] \frac{\mathrm{d}\omega}{(2\pi)^3} \tag{5}$$

Hence we identify the spectral phonon transmission function $\Xi(\omega) = t_\omega g(\omega)$ where $g(\omega) = \mathrm{d}k_x \mathrm{d}k_y$ is the projected phonon density of states in the non-periodic directions of the system.

We probe the spectral phonon transmission function $\Xi(\omega)$ by atomistic Greens Function (AGF) [1, 2] and the thermal conductance can be obtained by following the Landauer formula:

$$G = \int_0^{\omega_{max}} \Xi(\omega) \frac{\partial}{\partial T} \left(e^{\frac{\hbar\omega}{k_B T}} - 1\right)^{-1} \hbar\omega \frac{d\omega}{2\pi} \tag{6}$$

where ω and ω_{max} are the energy and the Debye frequencies. T refers to the mean temperature of the system, k_B and $\hbar$ represent the Boltzmann and the reduced Plancks constants, respectively. The transmission $\Xi(\omega)$ is obtained from a nonequilibrium Greens function approach as $\mathrm{Tr}[\Gamma_L G_s \Gamma_R G_s^+]$. The advanced and retarded Green functions G_s^+ and G_s can be deduced from

$$G_s = \left[(\omega + i\Delta)^2 I - K_s - \Sigma_L - \Sigma_R\right]^{-1} \tag{7}$$

where Δ is an infinitesimal imaginary part that maintains the causality of the Greens function and $\Sigma_L = K_{ab} g_L K_{ab}^+$, $\Sigma_R = K_{ab} g_R K_{ab}^+$ are the self-energies of the left and right leads, the "+" exponent indicating the Hermitian conjugation. The leads refer to the infinite stacking of graphene layers. Finally, g_L and g_R refer to the surface Greens functions of the left and the right leads, while K_s and K_{ab} are the force constant matrices derived from the potential, for the molecule and between neighboring graphene sheet, respectively. The expression of the transmission also includes $\Gamma_L = i(\Sigma_L - \Sigma_L^+)$ and $\Gamma_R = i(\Sigma_R - \Sigma_R^+)$.

SUPPLEMENTARY NOTE 5. ELECTRON TRANSPORT AND CONTRIBUTION TO THE CROSS-PLANE THERMAL CONDUCTANCE

Ab initio calculations were carried out using the quantum chemistry DFT code SIESTA [3]. All systems were first geometrically optimized in isolation, with a generalized local-density approximations (LDA) within Ceperley-Alder version (CA), double-zeta polarized basis set, 0.01 eV/Å force tolerance and 250 Ry mesh cutoff. The relaxed atomic structures can be seen in Supplemental Figures. Electron transmission coefficients were computed using the GOLLUM [4] code. To produce the conductance curves in Supplementary Figure 8, the transmission coefficient $T_{\mathrm{el}}(E_{\mathrm{F}})$ was calculated for each relaxed junction geometry, by first obtaining the corresponding Hamiltonian and then overlapping matrices with SIESTA and double-zeta polarized basis set. To produce conductance-trace curves, the transmission coefficient $T_{\mathrm{el}}(E_{\mathrm{F}})$ was calculated for each relaxed junction geometry and the conductance $G_{\mathrm{el}}/G0 = T_{\mathrm{el}}(E_{\mathrm{F}})$ was obtained by evaluating $T_{\mathrm{el}}(E_{\mathrm{F}})$ at the Fermi energy E_{F}.

As shown in Supplementary Figure 8(c) and (d), the presence of the molecule reduces the electron transmission by interrupting the $\pi - \pi$ stacking of the phenyl rings in the adjacent graphene flakes. This means that the silane molecule eliminates the electrical current leakage by isolating the graphene heat spreader and the electronic device.

SUPPLEMENTARY NOTE 6. GRAPHENE OXIDATION ON THE IN-PLANE AND CROSS-PLANE THERMAL TRANSPORT IN THE SUPPORTED GRAPHENE FILM

Heat transport in graphene oxide has been a hot topic recently [5–7]. Present studies on the thermal transport of graphene oxide [1-3] have all focused on the influence of oxidation on the in-plane thermal conductivity of the graphene oxide. However, none of them studied the cross-plane heat conduction and/or the impact of oxidation on the heat flow in the adjacent graphene sheets of a graphene oxide - graphene hybrid system. Hence, we indeed agree with our Reviewer to conduct in-depth calculations on this so-far undiscovered subject.

We clarify the impact of oxidation of the graphene substrate on the in-plane and cross-plane thermal transport in the supported graphene film. As is shown in Supplementary Figure 9(a), the functionalized graphene layer at the bottom is oxidized on both sides of the

functionalization. The procedure of our molecular dynamics simulation is the same as in the Ref. [1]. In Supplementary Figure 9(b)., we plot thermal conductivity κ of the graphene film and its thermal resistance R with the functionalized graphene oxide support versus the oxidation coverage.

For a very weak oxidation, the thermal resistance R of the graphene film and its oxidized substrate and its thermal conductivity κ are very close to that for a non-oxidized graphene support. As the oxygen coverage increases on the graphene support, the thermal resistance start to decrease. Such a reduction is due to the van-der-Waals (vdW) interaction between the oxygen atoms and the above graphene film, in addition to the direct graphene-graphene vdW interaction. Consequently, the basal-plane thermal conductivity of the graphene film decreases along with the increase of the oxidation coverage. Such a decrease is due to better thermal coupling between the graphene oxide support and the graphene film. However, as can be observed from Supplementary Figure 9(b), the reduction in both film-support thermal resistance and the basal-plane thermal conductivity remains weak, due to the weak contribution of the vdW forces between the oxidation and the graphene film above.

SUPPLEMENTARY NOTE 7. CALCULATION OF THERMAL RESISTANCE AND THERMAL CONDUCTIVITY USING EQUILIB- RIUM MOLECULAR DYNAMICS

Adaptive intermolecular reactive empirical bond order (AIREBO) potential [8] was used to simulate the graphene's C-C interactions. AIREBO potential describes the bonded C-C interactions ($r < 2\AA$, where r is the distance between two C atoms) with the reactive empirical bond order (REBO) potential of Brenner and uses Lennard-Jones (LJ) potential for the non-bonded Van der Waals C-C interactions ($2\AA < r <$ cutoff radius). The long-range electromagnetic and the short-range repulsive-attractive interactions in the molecule is taken into account through the ReaxFF potential, which uses distance-dependent bond-order functions to represent the contributions of chemical bonding to the potential energy [9]. The initial inter-plane distance of the graphene sheets is set to be $3.35\AA$. After the insertion of the molecule, the distance increases to $7.5\AA$.

By using EMD, the trajectories in the phase space, i.e., the time-dependent positions and velocities of the carbon atoms, are computed. The temperature T can be readily calculated

from the velocities of the atoms in the simulation domain by using the time dependent kinetic energies. The thermal resistance R between two adjacent graphene sheets with temperature difference ΔT could be calculated by the following equation [10]:

$$Rk_B = \int_0^\infty \frac{\langle \Delta T(0)\Delta T(t)\rangle}{\langle \Delta T(0)^2\rangle} dt \left(\frac{1}{N_1} + \frac{1}{N_2}\right) \tag{8}$$

where k_B is the Boltzmann constant, N_1 and N_2 refer to the number of degrees of freedom of the graphene subsystems sheets in interaction.

The in-plane thermal conductivity κ is calculated by the Green-Kubo formula:

$$\kappa = \frac{V}{k_B T^2} \int_0^\infty \langle J(0)J(t)\rangle dt \tag{9}$$

where V is the system volume, T refer to the equilibrium temperature of the system and $J(t)$ is the time-dependent in-plane heat current.

Periodic boundary conditions are applied in the in-plane directions and free boundary condition in the cross-plane direction of the graphene system. First, each super cell was relaxed at the simulation temperature to achieve zero in-plane stress. Then the systems were thermalized by using a Langevin heat bath. After the thermalization, MD runs with lengths equal to 600 ps and 1 ns were carried out in the microcanonical ensemble to sample the temperature and heat flux to be used in the calculation of the thermal resistance and thermal conductivity by following Eq. (8) and (9), respectively.

As an example of our computational procedure of the thermal resistance and thermal conductivity and the convergence of the MD simulations, Supplementary Figure 10a and 10b presents the time integrals of the temperature difference and heat flux autocorrelation function (ACF) from Eq. (8) and (9), respectively. The data points of the thermal resistance and thermal conductivity in Fig. 2 of the manuscript were obtained by averaging independent simulation trajectories (black curves in Supplementary Figure 10) until the ensemble average converged (red curves in Supplementary Figure 10). More than fifty independent simulations were performed for each data point Fig. 6. of the manuscript The error bars shown in Fig. 2 of the manuscript were defined based on the standard deviation of the averaged values.

One important remark has to be made about predicting thermal properties of graphene-based materials by using classical molecular dynamics. In classical MD simulations, all phonon modes below the Debye frequency ω_θ are equally excited. The phonon occupation

number writes,

$$n = \frac{k_B T}{\hbar\omega} \tag{10}$$

which is the high temperature limit $\hbar\omega \ll k_B T$ of the Bose-Einstein distribution, At room temperature $T = 300$ K, only vibrations until 6.3 THz are thermally excited according to BE statistics., whereas the Debye frequency of graphene is around 60 THz. In other words, to excite half of the graphene phonon spectrum, the temperature has to reach around 1500 K. Hence the classical phonon distribution overestimates the thermal conductivity of graphene at temperatures lower than 1500 K since it includes the specific heat of the high frequency phonons. On the other hand, the thermal conductivity of graphene is mainly contributed by the low frequency phonons of the flexural "ZA" mode. The high frequency phonons, especially the optical ones, do not contribute much to the overall thermal conductivity. Therefore the error in the estimation of the thermal conductivity of graphene introduced by using classical phonon distribution should be within a tolerable range.

SUPPLEMENTARY NOTE 8. THERMAL CONDUCTANCE OF A SINGLE SILANE MOLECULE

The total thermal conductance G_t of the junction comprises of the conductance G_m through a single molecule and the conductance G g through direct interaction of graphene sheets. Their relation writes simply,

$$G_t = G_g + G_m \tag{11}$$

G_g is proportional to the graphene contact area A, i.e., $G_g(A) \propto A$. We calculate with molecular dynamics the total thermal conductances for graphene systems of different contact area, as shown in Supplementary Figure 11. From the figure, we fit the data with a linear function and the y-intercept is the conductance of the molecule, i.e. $G_m = G_t(A = 0)$. Thus we get $G_m = 82$ pW K^{-1} .

SUPPLEMENTARY NOTE 9. CRITICAL LAYER NUMBER OF GRAPHENE FOR THERMAL PROPERTY SWITCH

We identify here the factors that determine the critical layer number for the thermal property change. These factors are I) number density of the functionalization molecules, II) functional groups of the molecule, and III) substrate of the graphene film.

- I. Molecule density.

  i) For a diluted molecule density, as shown in Supplementary Figure 12a, the ripples are formed not only on the graphene layer which is directly covalently bonded to the molecule, but also on the top layer which has no direct covalent bonds with the underlying layer. Such geometrical defects are phonon scatterers and in this case the two layers of graphene with functionalization has lower basal-plane thermal conductivity compared to that without. In this density range, the ripples are well separated from each other and increasing the molecule density will decrease linearly the phonon MFP. The critical number l_c for the trend switch will be higher than 2 since more graphene sheets are required to recover the flat conformation, i.e. $l_c > 2$. The precise layer number l_c will depend on the molecule density.

  ii) For a high molecule density, as shown in Supplementary Figure 12b, the ripples no longer remain separated and start to merge with their neighbours, recovering the flat surface in an extended area. Such merging hence recovers the interrupted phonon MFP and alleviates the detrimental influence of the separate ripples on the basal-plane thermal conductivity of graphene. In this case the two layers of graphene with functionalization have higher basal-plane thermal conductivity compared to that without functionalization, i.e. $l_c = 2$.

- II. Functional group of the molecule.

  To examine the effect of other functional groups, we use another functionalization molecule named HLK-5 which has been experimentally applied to reduce the thermal resistance between carbon nanotube (CNT) fillers and the polymer matrix [11]. The difference between the HLK-5 molecule and the amino-sliane used in the present study is that its amino group is doubly bonded to an aromatic ring of graphene/CNT. Such a double bond has a stronger bond strength than the single bond of the amino-silane

molecule, thus resulting in a more severe static graphene distortion. Therefore in this case the two layers of graphene with functionalization have lower basal-plane thermal conductivity compared to that without.

- III. Substrate of the graphene film.
  To investigate the effect of a different substrate on the critical number of layer, we use here a crystalline silica substrate to support the graphene film. The silica substrate could be easily functionalized with amino-silane molecules. The molecular dynamiscs simulation parameters are the same as in [12]. As can be seen from Supplementary Figure 14, the graphene sheets experience remarkable surface distortion when supported on a silica substrate. Such distorsion results from the strong lattice mismatch between the silica substrate and the graphene film. Previous studies show that when supported on an amorphous substrate, of the suspended graphene decreased by almost one order of magnitude, from 4000 $Wm^{-1}K^{-1}$ to 600 $Wm^{-1}K^{-1}$. Due to such severe distortion, two layers of graphene is not sufficient to recover the high basal-plane thermal conductivity, even with the intercalation of the functionalization.

SUPPLEMENTARY NOTE 10. PHONON RELAXATION TIME AND DISPERSION CURVE CALCULATIONS USING EQUILIBRIUM MOLECULAR DYNAMICS

We extract phonon liftetime $\tau(\mathbf{k})$ by using the normal mode decomposition technique. In the equilibrium molecular dynamics, the atomic positions and velocities are projected onto the normal mode coordinates of the system. The normal mode coordinates are then used to calculate the normal mode potential and kinetic energies.

The normal mode coordinate $q(\mathbf{k}, t)$ writes as a spatial Fourier Transform of the atomic displacements $\mathbf{u}_j(t)$ [13, 14]

$$q(\mathbf{k}, t) = \sum_j (m_j/N)^{1/2} e^{i\mathbf{k}\mathbf{r}_j^0} \mathbf{e}_j(\mathbf{k}) \mathbf{u}_j(t) \tag{12}$$

and its time derivative $\dot{q}(\mathbf{k}, t)$

$$\dot{q}(\mathbf{k}, t) = \sum_j (m_j/N)^{1/2} e^{i\mathbf{k}\mathbf{r}_j^0} \mathbf{e}_j(\mathbf{k}) \dot{\mathbf{u}}_j(t) \tag{13}$$

where j is the atom index, m_j, $\mathbf{r}_j^0$ and $\mathbf{e}_j(\mathbf{k})$ refer to the mass, the equilibrium position and the corresponding eigenvector of the atom j.

The potential and kinetic energies of the normal mode are

$$U(\mathbf{k},t) = \frac{1}{2}\omega(\mathbf{k})^2 q^\dagger(\mathbf{k},t)q(\mathbf{k},t) \tag{14}$$

and

$$T(\mathbf{k},t) = \frac{1}{2}\dot{q}^\dagger(\mathbf{k},t)\dot{q}(\mathbf{k},t) \tag{15}$$

The total energy of the normal mode is

$$E(\mathbf{k},t) = U(\mathbf{k},t) + T(\mathbf{k},t) \tag{16}$$

One can demonstrate that

$$\frac{< E(\mathbf{k},t)E(\mathbf{k},0) >}{< E(\mathbf{k},0)^2 >} = e^{-2\Gamma(\mathbf{k})t} \tag{17}$$

where $\Gamma(\mathbf{k})$ is the linewidth, equal to $1/[2\tau(\mathbf{k})]$. Thus, the lifetime can be approximated as

$$\tau(\mathbf{k}) = \int_0^\infty \frac{< E(\mathbf{k},t)E(\mathbf{k},0) >}{< E(\mathbf{k},0)^2 >}\mathrm{d}t \tag{18}$$

To calculate the phonon dispersion curve, we first compute the spectral energy density of the mode $\mathbf{k}$ which correponds to a double Fourier Transform of the atomic velocities in Eq. (13) to the reciprocal space of frequency and wave vector

$$E_{\mathrm{SED}}(\mathbf{k},\omega) = \left| \int_{-\infty}^{\infty} \dot{q}(\mathbf{k},t)\exp(-i\omega t)\mathrm{d}t \right|^2 \tag{19}$$

In a harmonic system, phonons do not scatter. Therefore their $E_{\mathrm{SED}}(\mathbf{k},\omega)$ and exhibit delta-function type peaks in vibrational spectrum. In real material systems, phonons scatter and thus have finite lifetime due to the anharmonic interactions, leading to the linewidth broadening and thus giving Lorentzian peak shapes, as shown in Supplementary Figure 15.

SUPPLEMENTARY NOTE 11. IN-PLANE THERMAL CONDUCTIVITY ENHANCEMENT DUE TO INCREASED RELAXATION TIME

With the presence of the molecule, the ZA phonon branch has a finite frequency value at the Brillouin zone center, i.e. $\omega(k = \Gamma) > 0$, as compared to the zero frequency for graphene

without the molecule. Such massive ZA branch reduces the phonon group velocity v_g in the frequency region near the zone center, which is susceptible to reduce the in-plane thermal conductivity if the phonon relaxation time τ is not changed, since the thermal conductivity writes $\kappa \propto C_v v_g^2 \tau$, where C_v is the specific heat per volume. However, as it can be seen in Fig. (6) of the manuscript, that the phonon relaxation time is substantially increased by the presence of the molecule over the entire phonon spectrum.

In order to clarify the competing effect of the increase phonon relaxation time and the decreased group velocity, we calculate the spectral in-plane thermal conductivity $\kappa(\omega)$ of the ZA branch by following the single-mode-relaxation-time approximation based on the kinetic theory,

$$\kappa(\omega) = C_v(\omega) v_g(\omega)^2 \tau(\omega) \tag{20}$$

where $C_v(\omega)$, $v_g(\omega)$ and $\tau(\omega)$ refer to the specific heat per volume, phonon group velocity and relaxation time extracted from Fig. (6) of the manuscript. The spectral thermal conductivity $\kappa(\omega)$ of the ZA branch is shown in Supplementary Figure 16. At low frequencies ($\omega/2\pi \leq 2.2$ THz), the substantially increased relaxation time compensates the effect of the reduction in the phonon group velocity v_g by the massive ZA branch due to the presence of the molecules. At larger frequencies, the in-plane thermal conductivity is enhanced mostly due to the increase relaxation time. This remains valid for the rest of the phonon branches.

SUPPLEMENTARY NOTE 12.DEPENDENCE OF IN-PLANE THERMAL CONDUCTIVITY OF GRAPHENE ON THE MOLECULE DEN- SITY

We clarify the thermal conductivity dependence on the number density ρ of the functionalization molecules. We identify three regions where the thermal conductivity demonstrates different behavior following the molecule number density:

- very diluted regime, $\rho \leq 0.01$ nm^{-2}: For a very low molecule density, the basal-plane thermal conductivity shows very slight reduction compared to the case "wo/ molecule", as shown in Supplementary Figure 17. In fact, when the number density of the molecule is further decreased (e.g. $\rho = 0.0025$ nm^{-2} corresponding to one single molecule on a 20 nm × 20 nm basal-plane area of graphene), the thermal conductivity

of the "w/ molecule" case equals to that of the case "wo/ molecule" with the precision of the error bar.

- Region I (diluted regime, $0.01\ \text{nm}^{-2} \leq \rho \leq 0.055\ \text{nm}^{-2}$): When the molecule density increases, the ripples formed on the graphene surface which are caused by the point defects on the lower graphene layer due to the covalent functionalization start to scatter phonons and reduce the phonon meam-free-path (MFP). Note that in this regime, the ripples are well separated from each other and increasing the molecule density will decrease linearly the phonon MFP.

- Region II (intermediate regime, $0.055 \leq \rho \leq 0.11\ \text{nm}^{-2}$): When ρ further in- creases, the ripples no longer remain separated and start to merge with their neighbours. Such merging recovers the interrupted phonon MFP and alleviates the detrimental influence of the separate ripples on the basal-plane thermal conductivity of graphene. In addition, the increase of the thermal resistance R of the supported graphene and its functionalized substrate starts to gain importance since the molecules reduces their van-der-Waals (vdW) interaction by intercalation. The heat channels through the molecules are weak compared to the direct graphene-graphene interlayer vdW interaction and therefore the overall cross-plane thermal resistance is increased. Such increased R will further favor the in-plane heat conduction thus enhancing the basal-plane thermal conductivity.

- Region III (dense regime, $\rho \geq 0.11\ \text{nm}^{-2}$): When the molecule density continues to increases, the molecules introduce numerous channels to transport heat in the cross-plane direction, whereas the thermal conductance of direct graphene-graphene interaction remains unchanged because the interlayer distance is kept stable by the molecules. Therefore the overall substrate-graphene thermal conductance is increased and the basal-plane thermal conductivity starts to fall.

In heat-spreader applications using chemical functionalization, the molecule density is generally large. In the work of T. Meier et al., a Self-Assembled Monolayer with a number density of $4.67\ \text{nm}^{-2}$(see manuscript Ref. [38]), which is in the region III of Fig. R22, were used to study the cross-plane heat transport from a silica substrate to a gold atomic-force microscope tip. Therefore, we present in Fig. 5. of the manuscript the basal-plane thermal

conductivity for $\rho \geq 0.11$ nm^{-2}.

SUPPLEMENTARY NOTE 13. RAMAN SPECTRA OF GBF

Raman spectroscopy was performed on the GBF before and after spin coating the functionalized graphene oxide (FGO) layer between 10-20 nm thick, as shown in where Supplementary Figure 18. The D band, G Band and 2D band of the Raman spectra was at 1334 cm^{-1}, 1577 cm^{-1} and 2657 cm^{-1}, respectively, before spin coating. The noise level is represented by D band, with the relatively intense D band signifying the presence of defects due to chemical reduction. In contrast, after the spin coating, the intensity ratio of I_D/I_G was 1.4, compared to that of 0.9 before spin coating, with this attributed to the presence of FGO on the GBF surface. The multilayered structure of the GBF is evidenced by $I_{2D}/I_G < 1$.

SUPPLEMENTARY NOTE 14. FTIR SPECTRA OF GBF

Fourier transform infrared spectroscopy (Spectrum Two, PerkinElmer) was conducted to study the functional groups on the GBF before and after spin coating with FGO. As shown in Supplementary Figure 19, before spin coating FGO, no significant peak is present between the range of 2600-3100 cm^{-1} and 600-1600 cm^{-1}. After spin coating, the peaks at 2884 cm^{-1} and 2929 cm^{-1} emerged, corrresponding to stretching vibrations of CH and CH^{-2} of the 3-Aminopropyl triethoxysilane (APTES) molecules. Moreover, peaks at 1034 cm^{-1} and 691 cm^{-1} indicate the presence of Si-O-Si and -Si-C- group, which provides more evidence for functionalization.

SUPPLEMENTARY REFERENCES

[1] N. Mingo and L. Yang, Phys. Rev. B **68,** 245406 (2003).

[2] J. S. Wang, N. Zeng, J. Wang, and C. K. Gan, Phys. Rev. E **75,** 061128 (2007).

[3] Soler, Jos M., et al. The SIESTA method for ab initio order-N materials simulation Journal of Physics: Condensed Matter **14, 11** 2745 (2002).

[4] J Ferrer, CJ Lambert, VM Garca-Surez, D Zs Manrique, D Visontai, L Oroszlany, R Rodrguez-Ferrads, I Grace, SWD Bailey, K Gillemot, Hatef Sadeghi, LA Algharagholy GOLLUM: a next-generation simulation tool for electron, thermal and spin transport New Journal of Physics **16, 9** 093029 (2014).

[5] X. Mu, X. Wu, T. Zhang, D. B. Go and T. Luo, Thermal Transport in Graphene Oxide From Ballistic Extreme to Amorphous Limit. Sci. Rep. 4, 3909 (2014).

[6] Lin S and Buehler M J. Thermal transport in monolayer graphene oxide: Atomistic insights into phonon engineering through surface chemistry. Carbon, 77, 351 (2014).

[7] Zhang H, Fonseca A F and Cho K. Tailoring thermal transport property of graphene through oxygen functionalization. The Journal of Physical Chemistry C, 118, 1436 (2014).

[8] S. J. Stuart, A. B. Tutein, and J. A. Harrison, J. Chem. Phys. **112**(14), 6472 (2000).

[9] Chenoweth, van Duin and Goddard, Journal of Physical Chemistry A, 112, 1040-1053 (2008).

[10] A. Rajabpour and S. Volz, J. Appl. Phys. **108**(9), 094324 (2010).

[11] Ni, Y., Le Khanh, H., Chalopin, Y., Bai, J., Lebarny, P., Divay, L., and Volz, S. Highly efficient thermal glue for carbon nanotubes based on azide polymers. Applied Physics Letters, 100(19), 193118 (2012).

[12] Y Ni, YA Kosevich, S Xiong, Y Chalopin and S Volz, Substrate-induced cross-plane thermal propagative modes in few-layer graphene. Physical Review B 89 (20), 205413 (2014)

[13] M. T. Dove, *Introduction to lattice dynamics* (Cambridge University Press, Cambridge, 1993).

[14] J. E. Turney, E. S. Landry, A. J. H. McGaughey, and C. H. Amon, Phys. Rev. B **79,** 064301 (2009).

[15] T. Meier, F. Menges, P. Nirmalraj, H. Hölscher, H. Riel,1 and B. Gotsmann. Length-Dependent Thermal Transport along Molecular Chains. Phys. Rev. Lett. **113,** 060801 (2014).
